# Multi-functional genome-wide CRISPR system for high throughput genotype–phenotype mapping

**DOI:** 10.1038/s41467-019-13621-4

**Published:** 2019-12-19

**Authors:** Jiazhang Lian, Carl Schultz, Mingfeng Cao, Mohammad HamediRad, Huimin Zhao

**Affiliations:** 1 Department of Chemical and Biomolecular Engineering, Carl R. Woese Institute for Genomic Biology, University of Illinois at Urbana-Champaign, Urbana, IL 61801 USA; 2 Key Laboratory of Biomass Chemical Engineering of Ministry of Education, College of Chemical and Biological Engineering, Zhejiang University, 310027 Hangzhou, China; 3 Departments of Chemistry, Biochemistry, and Bioengineering, University of Illinois at Urbana-Champaign, Urbana, IL 61801 USA; 4 Present Address: Lifefoundry Inc., 60 Hazelwood Dr., Champaign, IL 61820 USA

**Keywords:** Metabolic engineering, Synthetic biology, CRISPR-Cas systems

## Abstract

Genome-scale engineering is an indispensable tool to understand genome functions due to our limited knowledge of cellular networks. Unfortunately, most existing methods for genome-wide genotype–phenotype mapping are limited to a single mode of genomic alteration, i.e. overexpression, repression, or deletion. Here we report a multi-functional genome-wide CRISPR (MAGIC) system to precisely control the expression level of defined genes to desired levels throughout the whole genome. By combining the tri-functional CRISPR system and array-synthesized oligo pools, MAGIC is used to create, to the best of our knowledge, one of the most comprehensive and diversified genomic libraries in yeast ever reported. The power of MAGIC is demonstrated by the identification of previously uncharacterized genetic determinants of complex phenotypes, particularly those having synergistic interactions when perturbed to different expression levels. MAGIC represents a powerful synthetic biology tool to investigate fundamental biological questions as well as engineer complex phenotypes for biotechnological applications.

## Abstract (web summary)

Genome-scale engineering is generally limited to single methods of alteration such as overexpression, repression or deletion. Here the authors present a tri-functional CRISPR system that can engineer complex synergistic interactions in a genome-wide manner.

## Introduction

Functional profiling of genotype–phenotype relationships has broad applications in both fundamental biology and biotechnology, such as to decipher the genetic determinants of microbial pathogenesis and construct cell factories with maximal production of the desired metabolites^1^. Nevertheless, our understanding of the complexity of the cellular network is rather limited. For example, only about 1000 genes are included in the most advanced genome-scale metabolic models of *Saccharomyces cerevisiae*, although there are more than 6000 genes in the genome of this most well-studied eukaryote^2,3^. In other words, most genes have not been clearly mapped into biological pathways or phenotypic traits. Therefore, the identification of genetic determinants and the elucidation of their synergistic interactions remain the biggest challenges for understanding and engineering complex phenotypes.

Genome-scale engineering that can create libraries of genetic variants covering all the possible genes provides a promising strategy for functional genomics^1,4^, overcoming our limited knowledge of biocomplexity. Recently, the clustered regularly interspaced short palindromic repeats (CRISPR)/CRISPR-associated (Cas) system has revolutionized the genome engineering field and was recently adopted for genome-scale engineering^5–7^. By introducing pooled or arrayed guide RNA (gRNA) libraries that can target all the genes of a specific organism, CRISPR has enabled the construction of genome-wide deletion (CRISPRd) libraries in bacteria^8^, yeasts^9^, and mammalian cell lines^10^. Moreover, by using the nuclease-deficient CRISPR protein (dCas), genome-scale transcriptional activation (CRISPRa)^11–13^ and interference (CRISPRi)^13–15^ have been demonstrated in various hosts as well. Nevertheless, there is still no report on the development of a multi-functional genome-scale CRISPR system, and the genetic determinants were identified using a single type of modulation (activation, interference, or deletion). Recently, the guidelines for genome-scale CRISPR knockout and activation screening were provided independently. However, due to the use of the same Cas protein to create both libraries, interactions between the deletion and activation targets could not be explored^16^. In other words, the genotypic diversity created by existing methods is not comprehensive, as both upregulation and downregulation of multiple targets are generally required to engineer the desired phenotype^2,3^. For example, overexpression of the mevalonate pathway and repression of the downstream ergosterol biosynthetic pathway worked synergistically for the carotenogenesis phenotype^17^.

Considering the demands for genetic manipulation of multiple targets with different modes of alteration and the availability of CRISPR modules, there is a growing interest in the development of multi-functional CRISPR systems for both fundamental studies and biotechnological applications. The first dual-functional CRISPR system was developed by the scaffold RNA (scRNA) strategy, where aptamer sequences (i.e. MS2) were fused to the gRNA scaffold to recruit transcriptional regulators to dCas9 via aptamer–RNA-binding protein interactions. The specific interaction between aptamer and RNA-binding protein enabled CRISPRa and CRISPRi to work independently in the same cell^18^. Simultaneous gene deletion and transcriptional activation was achieved using Cas-activator fusion proteins via gRNA engineering, truncated gRNAs for CRISPRa and full-length gRNAs for CRISPRd^19–21^. By taking advantage of the binding position effect, a dCas9-based activator was repurposed to function as a dual-mode activator/repressor, which could block transcription initiation and elongation when targeting the core promoter and coding sequence regions (CRISPRi) and served as a transcriptional activator when targeting the upstream sequences of the core promoter (CRISPRa)^22^. In our previous studies, we developed a tri-functional CRISPR system (CRISPR-AID) using three orthogonal Cas proteins to integrate gene activation, interference, and deletion into the same host. In the CRISPR-AID system, the catalytically inactive Cas12a from *Lachnospiraceae bacterium* fused with an activation domain (dLbCas12a-VP) was used for CRISPRa, the nuclease-deficient Cas9 from *Streptococcus pyogenes* fused with a repression domain (dSpCas9-RD1152) for CRISPRi, and the catalytic Cas9 from *Staphylococcus aureus* (SaCas9) for CRISPRd^8^. Notably, none of these multi-functional CRISPR systems has been attempted at the whole genome scale, limiting their wide applications in high throughout functional genomics and complex phenotype engineering.

In the present study, we develop a multi-functional genome-wide CRISPR (MAGIC) system for high throughput genotype–phenotype mapping. By combining CRISPR-AID and array-synthesized oligo pools, we create genome-scale gain-of-function, reduction-of-function, and loss-of-function libraries, which represents, to the best of our knowledge, one of the most comprehensive and diversified genomic libraries ever reported in yeast. MAGIC is then used to identify previously uncharacterized genetic determinants of complex phenotypes, i.e. furfural tolerance and protein surface display, either iteratively (iMAGIC) or simultaneously (sMAGIC). Finally, we explore the synergistic interactions among MAGIC-identified targets when regulated to different expression levels.

## Results

### Design of MAGIC for high throughput functional genomics

**Fig. 1:**
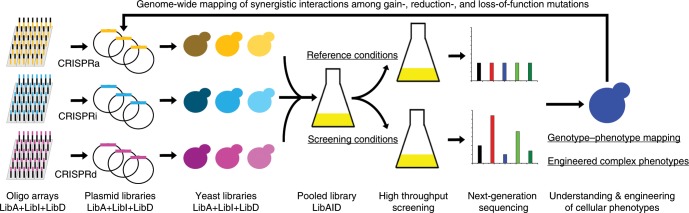
The MAGIC pipeline for genome-wide mapping genotype–phenotype relationships. Guide sequences for genome-scale activation (orange), interference (light blue), and deletion (magenta) were synthesized as arrayed oligos on DNA chip and cloned into the corresponding gRNA expression plasmids using Golden-Gate Assembly. The iMAGIC library was constructed by transforming the pooled plasmid libraries into the CRISPR-AID integrated yeast strain, and subject to growth enrichment under various conditions or high throughput screening. The enrichment and depletion of guide sequences were profiled using next-generation sequencing. The iMAGIC workflow can be iterated to better understand and engineer complex phenotypes.

In our previous study, we have constructed the CRISPR-AID system. This multi-functional genome engineering technology enabled the combinatorial optimization of many pre-defined targets for the construction of optimal yeast cell factories^8^. To further develop the MAGIC system, we designed and constructed three genome-scale gRNA-expressing plasmid libraries from pools of array-synthesized oligos, each for upregulating, downregulating, and deleting all the genes in the yeast genome, respectively. Transforming the plasmid libraries into the CRISPR-AID-integrated *S. cerevisiae* strain^8^ resulted in the construction of the MAGIC library (Fig. 1), where a full spectrum of expression profiles were achieved for all defined genes throughout the whole genome. The MAGIC library, which represents one of the most comprehensive and diversified genomic libraries ever reported in yeast, was grown with or without a certain stress or subject to high throughput screening to associate our target phenotypes with their strongest genetic determinants in the yeast genome. The unique guide sequence in each plasmid serves as a genetic barcode for high throughput phenotyping by next-generation sequencing (NGS). Genotype–phenotype relationships can be mapped by tracking the enrichment or depletion of guide sequences, and the synergistic or additive interactions among gain-of-function, reduction-of-function, and loss-of-function mutations can be identified in an iterative and genome-wide manner.

To facilitate iterative MAGIC screening, we characterized several genomic loci for SaCas9-assisted and marker-less integration of gRNA expression cassettes. Previously reported integration loci^23^ were chosen, which were flanked by highly expressed essential genes to enable efficient and stable expression of heterologous genes and pathways. Ten gRNA plasmids based on SaCas9 were constructed to integrate heterologous cassettes into X2, X3, X4, XI1, XI2, XI3, XII1, XII2, XII4, and XII5 loci, respectively (Supplementary Table 1). The gRNA-targeting efficiency was tested by transforming the gRNA plasmid without any donor to repair the double strand break: efficient gRNAs should result in no surviving colonies. The integration efficiency and gRNA expression levels were evaluated by co-transforming the reporter strain (bAID-RV) with the gRNA plasmid, as well as its corresponding linear donor fragment, which contained a gRNA expression cassette to activate the expression of mCherry or to repress the expression of mVenus. Eight colonies were randomly picked to measure the change in fluorescence intensities. As shown in Supplementary Fig. 1 and Supplementary Table 1, X3, X4, XI1, XI3, XII2, XII4, and XII5 together with their corresponding gRNAs were chosen for CRISPR-assisted and marker-less integration of gRNA expression cassettes.

### Design and construction of the MAGIC libraries

To create the MAGIC library, we firstly obtained and ranked all possible guide sequences targeting all ORFs and RNA genes (rRNAs, tRNAs, snRNAs, snoRNAs, and ncRNAs) using previously described criteria and empirical experiences^8,9^ (Supplementary Table 2). To enable genome-scale gene disruption, the homologous recombination donor was integrated to the 5′-end of the targeting sequences^9^. Homology-directed repair resulted in the deletion of 28 bp nucleotides in the coding sequences, including both the targeting sequences and the protospacer adjacent motif sequences (Supplementary Fig. 2). Different from CRISPRd, the gRNA-binding sites relative to the transcriptional starting sites can be equally important as the guide sequences for CRISPRa and CRISPRi^8,13^. Therefore, the following criteria were included to rank the guide sequences: targeting efficiency, targeting position, GC content, and off-target score. The guide sequences containing polyT, polyG, and *Bsa*I sites were excluded. In addition, to make the genome-scale libraries more diversified, we only kept the top-ranked guide if multiple guide sequences were clustered together. We validated the ranking criteria using the previously designed gRNAs^8^ with high efficiency (Supplementary Table 3). For most of the targets, we selected six top-ranked guide sequences for the CRISPRa and CRISPRi libraries, while four for the CRISPRd library. On average, ~98% of the designed gRNAs showed high scores (Supplementary Fig. 3). We also included 100 randomly generated guide sequences as negative controls in each library. Adapters were added to both ends of these oligos for cloning purposes (Supplementary Table 4). In summary, we designed and synthesized 37,817, 37,870, and 24,806 unique guide sequences for the CRISPRa, CRISPRi, and CRISPRd libraries, respectively (Table 1 and Supplementary Table 5). All designed guide sequences with scores were summarized in Supplementary Data 1–3.

**Table 1:** Construction and characterization of the iMAGIC plasmid library.

	LibA	LibI	LibD
CRISPR protein	dLbCas12a-VP	dSpCas9-RD1152	SaCas9
Length of gRNA^a^	20 + 23 bp	20 + 82 bp	121 + 127 bp
No. of guides	37,817	37,870	24,806
Fold coverage^b^	~133×	~106×	~121×
Mapping ratio	~87.7%	~86.8%	~72.6%
gRNA coverage	~99.9%	100%	~88.9%
Gene coverage^c^	100%	100%	~98.3%

^a^The length of guide (underlined) and structural sequences

^b^Calculated as estimated library size/number of guide sequences

^c^At least one guide for each gene

The pooled oligonucleotides were amplified by PCR and cloned into the corresponding gRNA expression plasmids. The Golden-Gate Assembly efficiency was estimated to be nearly 100% by randomly genotyping 14 clones for each library. We sequenced the plasmid libraries and found that ~87% of the CRISPRa and CRISPRi libraries and ~73% of the CRISPRd libraries had the correct guide sequences. The lower mapping ratio of the CRISPRd library should result from higher synthesis error rate for longer oligos. As a result, more than 99.9% of all gRNAs and genes were covered in the CRISPRa and CRISPRi plasmid libraries, while there was at least one gRNA for ~98% of the yeast genes in the CRISPRd library (Table 1). The coverage of the genome-scale CRISPR-AID libraries was significantly higher than the previously reported cDNA-based genome-scale libraries^24^. We then created the iMAGIC library by transforming the three genome-scale plasmid libraries into the CRISPR-AID strains. The diversity of the iMAGIC library was evaluated by randomly genotyping 50 colonies of the CRISPRa yeast library (Supplementary Table 6).

### Validation of iMAGIC for engineering complex phenotypes

**Fig. 2:**
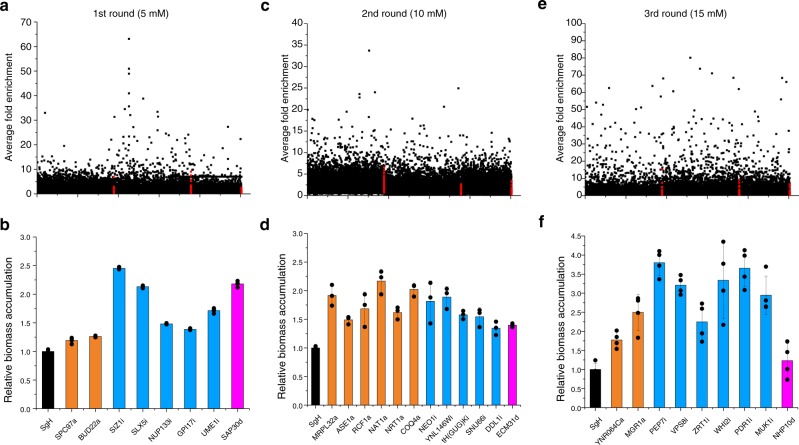
Iterative MAGIC enabled genome-wide mapping of furfural tolerance in yeast. The iMAGIC library was subject to iterative rounds of screening under gradually increased furfural concentration, 5, 10, and 15 mM for the first **a**, **b**, second **c**, **d**, and third **e**, **f** round of iMAGIC screening, respectively. The guide sequences of the enriched libraries were profiled **a**, **c**, **e** using next-generation sequencing and the top hits were verified **b**, **d**, **f** under the corresponding screening condition. The red dots represented the control guide sequences. Orange bars represented activation targets, light blue for repression, and magenta for deletion. Error bars represent the mean ± s.d. of biological triplicates. The source data for figures **b**, **d**, and **f** are provided as a Source Data file.

Next, we sought to use iMAGIC to identify genetic determinants of complex phenotypes, such as furfural tolerance and protein surface display. We screened the iMAGIC library in the presence of 5 mM furfural and observed many enriched guide sequences as compared to that under the reference conditions. Notably, the control guide sequences were not enriched, indicating the association of the enriched guide sequences with furfural stress (Fig. 2a). Among those highly enriched guides, *SIZ1i* (referring to *SIZ1* interference and the same afterwards) and *SAP30d* have been reported as furfural tolerance-related targets via genome-wide CRISPRd screening in *S. cerevisiae*^9,25^, while *SLX5i*, *NUP133i*, *GPI17i*, and *UME1i* were only identified in the present study (Fig. 2b). The identification of both known and unreported genetic targets suggests the effectiveness and power of iMAGIC for genome-wide profiling. Interestingly, Siz1p (E3 small ubiquitin-related modifier (SUMO)-protein ligase)^25^ and Slx5p^26^ (a subunit of the Slx5-Slx8 SUMO-targeted ubiquitin ligase complex) are both involved in ubiquitin-mediated protein degradation; Sap30p^27^ and Ume1p^28^ are both components of the Rpd3L histone deacetylase complex (Supplementary Table 7). These results highlight the roles of protein degradation and histone modification in furfural tolerance. As *SIZ1i* improved furfural tolerance the most, we constructed strain R1 by integrating the *SIZ1i* expression cassette into the X4 locus of the genome (Supplementary Table 1).

We then performed a second round of iMAGIC screening and enriched several additional guide sequences, which in combination with *SIZ1i* could further increase the growth rate in the presence of 10 mM furfural (Fig. 2c). Notably, none of the targets has been ever reported in association with furfural tolerance. Among those highly enriched guides, we found several targets related to mitochondrial functions. For example, Mrpl32p^29^ is a component of the large subunit of the mitochondrial ribosome, Rcf1p^30^ is a subunit of the cytochrome c oxidase, Coq4p^31^ is a mitochondrial protein involved in Coenzyme Q biosynthesis, Ddl1p^32^ is a mitochondrial located phospholipase associating with the remodeling of mitochondrial phospholipids, while Nat1p^33^ is a subunit of the protein acetyltransferase and was found to be an important element of mitophagy (Supplementary Table 7). We speculated that enhanced supply of ATP should be beneficial to tackle furfural stress. The repression of an uncharacterized ORF (*YNL146W*) and two RNAs (*SNU66* and a histidine tRNA gene) also improved furfural tolerance (Fig. 2d and Supplementary Fig. 4). Interestingly, none of the second round targets (i.e. *YNL146Wi*, *MRPL32a*, *RCF1a*, and *NAT1a*) improved furfural tolerance alone (Supplementary Fig. 5), indicating a dependence on and possibly synergistic interaction with *SIZ1i*. Then we used the *NAT1a* and *SIZ1i*-integrated strain (R2) as the parent strain for the third round of genome-wide screening and continued to observe highly enriched guide sequences (Fig. 2e). *PDR1i* was the optimal hit to improve furfural tolerance when integrated into the chromosome together with *SIZ1i* and *NAT1a* (R3, Fig. 2f and Supplementary Fig. 6). Pdr1p^34^ is a transcriptional factor that negatively regulates the expression of pleiotropic drug-resistance genes (i.e. *PDR5*). Thus, *PDR1i* could increase the expression of *PDR5* to export furfural out of the cell, leading to improved furfural tolerance.

**Fig. 3:**
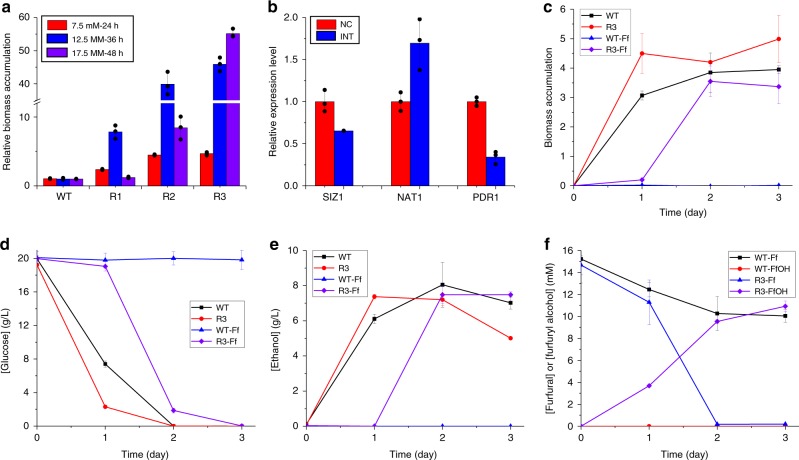
iMAGIC for the construction of a furfural tolerant yeast strain. **a** Furfural tolerance of the engineered strains identified in each round of iMAGIC screening, R1, R2, and R3. The cell densities of the engineered strains were normalized to the wild-type (WT) strain under the specified conditions (red bars for 7.5 mM furfural, blue for 12.5 mM, and purple for 17.5 mM). **b** Verification of gain-of-function and reduction-of-function mutations by qPCR. The expression level of each target (*SIZ1*, *NAT1*, and *PDR1*) was compared before (NC, red) and after (INT, blue) CRISPRa or CRISPRi cassette integration. Fermentation profiles including cell density **c**, glucose consumption **d**, ethanol production **e**, as well as furfural and furfuryl alcohol (FfOH) concentration **f** of WT (black square and blue triangle) and R3 (red circle and purple diamond) in synthetic medium with (blue triangle and purple diamond) or without (black square and red circle) the supplementation of 17.5 mM furfural (Ff). A single colony of WT or R3 was inoculated into 3 mL SED/G418 medium and cultured until saturation, which was then transferred into 50 mL fresh SED/G418 medium with or without the supplementation of 17.5 mM furfural in a 250 mL un-baffled shaker flask. Fermentation was performed under oxygen-limited conditions (30 °C and 100 rpm), and samples were taken every 24 h. The decrease of furfural concentration in WT might result from evaporation, as no growth and furfuryl alcohol production were observed. Notably, the cell density (biomass accumulation) in **f** was determined by measuring the absorbance at 600 nm using a UV–vis spectrometer. Error bars represent the mean ± s.d. of biological triplicates. The source data are provided as a Source Data file.

After three rounds of genome-scale engineering, we not only profiled genetic determinants of furfural tolerance, but also obtained an engineered strain showing ready growth at high furfural concentrations. As shown in Fig. 3a, the engineered strains grew much faster than the control strain, with more significant effect observed at higher furfural concentrations. Quantitative PCR (qPCR) confirmed the desired genome modification, including the interference of *SIZ1*, activation of *NAT1*, and interference of *PDR1* (Fig. 3b). We also compared the fermentation performance of the wild-type (WT) and the engineered (R3) strains (Fig. 3c–f). In the absence of furfural, these strains showed comparable fermentation performance. On the contrary, when 17.5 mM furfural was supplemented, the control strain failed to grow after 6 days of culture (Fig. 3c), while R3 was able to consume most of glucose in 2 days (Fig. 3d). More importantly, the final concentration of ethanol was comparable to the control strain under furfural-free conditions (Fig. 3e), indicating that the central metabolism of our engineered yeast strain was not significantly changed. The improved furfural tolerance was accompanied with the reduction of furfural to the less toxic furfuryl alcohol (Fig. 3f), a mechanism consistent with previous studies^25^.

Besides furfural tolerance, we also demonstrated the application of iMAGIC for the functional profiling of another complex phenotype, yeast surface display of recombinant proteins (Supplementary Fig. 7). Using the *Trichoderma reesei* endoglucanase (EGII)^8,24^ as an example, *HOC1d* was the highest enriched target to enhance protein secretion and surface display levels, followed by *UBP3i* and *MNN9i*. Hoc1p and Mnn9p are both subunits of the Golgi mannosyltransferase complex, the disruption of which minimized protein super-glycosylation and enhanced protein secretion^35^. Ubp3p^36^ is thiol-dependent ubiquitin-specific protease and its downregulation should enable higher protein stability and abundance (Supplementary Table 7). The bAID-EG-*HOC1d* strain was subject to a second round of iMAGIC screening, with *NUP157i* and *PDI1a* identified as the best targets. Pdi1p (protein disulfide isomerase) is essential for disulfide bond formation in secretory proteins and its overexpression has been found to work synergistically with the downregulation of mannosyltransferase encoding genes (i.e. *MNN9*)^8^, while the effect of *NUP157i* on protein secretion and display is still unknown.

### Synergistic interactions among iMAGIC-identified targets

**Fig. 4:**
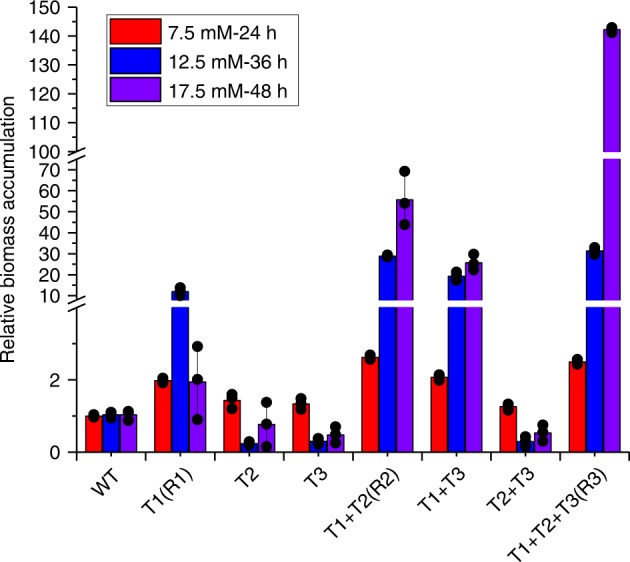
Synergistic interactions among iMAGIC identified targets (T). Single (T1, T2, and T3), double (T1 + T2, T1 + T3, and T2 + T3), and triple (T1 + T2 + T3) mutants were constructed to investigate the synergistic interactions among *SIZ1i*, *NAT1a*, and/or *PDR1i* for enhanced tolerance against furfural with a final concentration of 7.5 mM (red), 12.5 mM (blue), and 17.5 mM (purple). Error bars represent the mean ± s.d. of biological triplicates. The source data are provided as a Source Data file.

Finally, we asked whether there were synergistic interactions among the genetic determinants identified in iterative rounds of iMAGIC screening. Thus, we constructed single (T1 for *SIZ1i*, T2 for *NAT1a*, and T3 for *PDR1i*), double (T1 + T2, T1 + T3, and T2 + T3), and triple (T1 + T2 + T3) mutants and compared their tolerance against different concentrations of furfural. As shown in Fig. 4, the second and third round hits, alone (T2 or T3) or in combination (T2 + T3), marginally improved furfural tolerance in the reference strain. In other words, T2 and T3 only demonstrated furfural-tolerant phenotypes when combined with T1, demonstrating a synergistic interaction between *NAT1a* and *SIZ1i* as well as *PDR1i* and *SIZ1i*. Notably, T1 + T3 also endowed higher furfural tolerance than T1 and T3, particularly at high furfural concentrations. Therefore, there might be synergistic or additive effects between *NAT1a* and *PDR1i* in the *SIZ1i* background strain. Our results highlighted the effectiveness of iterative rounds of genome-wide screening in understanding and engineering of complex phenotypes.

### Exploration of genome-wide interactions simultaneously

**Fig. 5:**
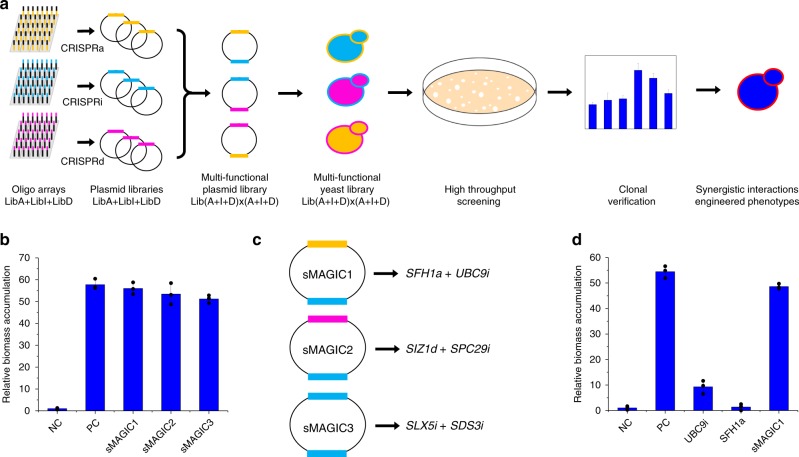
Engineering of furfural tolerance with sMAGIC. **a** The sMAGIC pipeline for furfural tolerance engineering. The sMAGIC plasmid library was constructed by assembling two genome-scale CRISPR-AID (LibA + LibI + LibD) into the same vector [Lib(A + I + D)×(A + I + D)] using Golden-Gate Assembly. The sMAGIC yeast library was constructed by transforming the sMAGIC plasmid library into the bAID strain and subject to colony-size-based high throughput screening on agar plates. **b** Furfural tolerance of the engineered strains identified by sMAGIC screening. The strains containing an empty vector (NC) and *SIZ1i*-*NAT1a* (PC) were included as a negative control and a positive control, respectively. **c** Sequencing results of top clones identified by sMAGIC screening, including sMAGIC1 (*UBC9i*-*SFH1a*), sMAGIC2 (*SIZ1d*-*SPE29i*), and sMAGIC3 (*SLX5i*-*SDS3i*). **d** Single (*UBC9i* or *SFH1a*) and double (*UBC9i*-*SFH1a*) mutants were constructed to investigate the synergistic interactions between *SFH1a* and *UBC9i* for enhanced tolerance against furfural (10 mM). Error bars represent the mean ± s.d. of biological triplicates. The source data for figures **b** and **d** are provided as a Source Data file.

Although iMAGIC was successfully demonstrated to identify genome-engineering targets with synergistic or additive interactions in an iterative and high throughput manner, only the top target was chosen for further studies. For example, the *SIZ1i*-integrated strain was subject to a second round of iMAGIC screening and genetic determinants showing synergistic interactions with *SIZ1i* were explored on a genome scale. However, our previous combinatorial optimization efforts indicated that the optimal combination does not necessarily include the best target when tested individually^8^. Therefore, to further explore the potential of the multi-functional CRISPR system, two MAGIC plasmid libraries were pieced together in the same vector using Golden-Gate Assembly (Fig. 5a and Supplementary Fig. 8), enabling the identification of synergistic interactions among gain-of-function, reduction-of-function, and loss-of-function targets on a genome scale simultaneously (sMAGIC, Fig. 5a). The Golden-Gate Assembly efficiency was estimated to be nearly 100% via diagnostic PCR. The diversity of the sMAGIC library was evaluated by randomly genotyping 40 clones (Supplementary Table 8). Due to the length of the double-gRNA cassettes, instead of growth enrichment followed by NGS, the sMAGIC yeast library strains were directly spread to the selective agar plates containing 10 mM furfural. Via genotyping of the largest colonies, we obtained a few previously unidentified combinations conferring furfural tolerance comparable to that of the second round iMAGIC-screened mutant (*SIZ1i*-*NAT1a*), including *SFH1a*-*UBC9i*, *SIZ1d*-*SPC29i*, and *SLX5i*-*SDS3i* (Fig. 5b, c). Notably, several of these gRNAs were also found to be highly enriched in the first round of iMAGIC screening, such as the down-regulation of *SIZ1*, *SLX5*, and *SDS3*. Among the previously unidentified combinations, the engineered strain with *SFH1* activation and *UBC9* interference was the most interesting. Sfh1p^37^ is a component of the RSC (remodeling of the structure of chromatin) chromatin remodeling complex, while Ubc9p^38^ is a SUMO-conjugating enzyme (Supplementary Table 7). Surprisingly, *SFH1* activation or *UBC9* interference alone only marginally or slightly improved furfural tolerance (Fig. 5d), indicating a synergistic interaction between *SFH1a* and *UBC9i*. Further studies are required to elucidate the detailed mechanism of *SFH1a*-*UBC9i*, particularly the synergistic interaction in improving furfural tolerance.

## Discussion

Compared with the traditional genome-scale engineering strategies, such as cDNA overexpression libraries^39^ and knock out collections^40^, CRISPR-based technology offers a more flexible alternative for constructing a genome-wide set of mutants under different strain backgrounds. Although there are prior CRISPR-enabled genome-scale engineering attempts, the genotypic diversity is only limited to the targets that share the same type of genomic alteration. To address this limitation, we developed MAGIC for mapping synergistic or additive interactions among overexpression, repression, and deletion targets in a genome-wide manner in *S. cerevisiae*. Taking the furfural-tolerant phenotype for example, the genome-wide RNAi technology (RAGE) failed to identify additional targets after one round screening with 5 mM furfural^25^, and another genome-scale CRISPRd system (CHAnGE) could not obtain enriched targets after two rounds of screening at 10 mM furfural^9^, while iMAGIC continued to enrich additional genetic determinants even after third rounds of screening at 15 mM furfural. Although screened under the same conditions (10 mM furfural and two rounds of evolution), the iMAGIC-engineered strain (*SIZ1i*-*NAT1a*) performed much better than the CHAnGE-modified strain (*SIZ1d*-*LCB3d*) (Supplementary Fig. 9). In other words, MAGIC not only identified more genetic determinants of furfural tolerance, but also engineered more furfural tolerant strains. Rational metabolic engineering strategies have also been employed to engineer furfural tolerance and the best performance in a laboratory yeast strain was achieved by overexpression of *TPS1* and *ARI1*, as well as the deletion of *NTH1* (*TPS1a-ARI1a*-*NTH1d*)^41^. As shown in Supplementary Fig. 10, the iMAGIC-engineered strain R3 (*SIZ1i*-*NAT1a-PDR1i*) demonstrated much higher furfural tolerance under the reported growth conditions (YPD medium with 30 mM furfural). These results demonstrated the necessity of combinatorial optimization and the power of MAGIC.

Recently, cDNA overexpression and RNA interference (RNAi) was combined to achieve combinatorial genome-scale engineering of complex phenotypes in yeast^24^. Both strategies enable the exploration of the gain-of-function and reduction-of-function combinations that work synergistically or additively to improve the desired phenotypes. Nevertheless, MAGIC not only introduces a third mode of genome engineering (gene deletion), but also offers several advantages of the CRISPR system. For example, MAGIC is less biased than the cDNA library, as all the MAGIC cassettes have the same or similar size to minimize cloning and transformation bias. More importantly, MAGIC represents one of the most comprehensive libraries ever created, with an average of > 99% coverage of all ORFs and RNA genes for genome-wide overexpression, repression, and deletion (Table 1). In comparison, the cDNA-based library covers ~92% of all ORFs^24^, as not all genes will be expressed under a given condition and RNA genes will not be included. In addition, the regulatory mechanisms are different, CRISPRi blocks transcription in the nucleus while RNAi affects mRNA stability and translation in the cytosol, and CRISPRi is generally believed to demonstrate higher gene repression efficiency than RNAi^13^.

The synergistic interactions among gain-of-function, reduction-of-function, and loss-of-function targets were identified either iteratively (iMAGIC) or simultaneously (sMAGIC). While iMAGIC has the advantages of high library coverage for functional genomics, its major limitation lies in the fact that only one perturbation (either activation, interference, or deletion) was made in each round. On the contrary, sMAGIC was established to identify synergistic interactions simultaneously, with two perturbations made to each strain as demonstrated in the present study. Theoretically, the sMAGIC library covered all the possible genomic perturbation combinations of LibA–LibA, LibA–LibI, LibA–LibD, LibI–LibI, LibI–LibD, and LibD–LibD. However, due to the limitations in transformation efficiency (~10^6^–10^7^ for yeast transformation), only ~0.01% to ~0.1% of the total combinations (~10^5^ × 10^5^ = 10^10^) were covered in the sMAGIC yeast library. The low library coverage might explain the failure to obtain any better gRNA combinations enabling higher furfural tolerance than the second round iMAGIC mutant (*SIZ1i*-*NAT1a*). Nevertheless, we could identify the combinations with synergistic interactions beyond the reach of iMAGIC. For example, neither *SFH1a* nor *UBC9i* were found to be highly enriched in the iMAGIC screening and we would never be able to identify the *SFH1a*–*UBC9i* combination in improving furfural tolerance using iMAGIC. In addition, we obtained several mutant strains with similar performance as the *SIZ1i*-*NAT1a* strain (R2) in a single round of screening, indicating sMAGIC as a powerful and yet time-saving tool for strain engineering. Due to the extremely low coverage, sMAGIC is not suitable for functional genomics and high throughput genotype–phenotype mapping. Overall, iMAGIC is more suitable for functional genomic studies, while sMAGIC has advantages in practical applications in strain engineering.

It is possible that MAGIC can be adopted for genome-scale engineering of higher eukaryotic organisms. For example, several orthogonal CRISPR proteins have been functionally characterized^42^ and genome-scale CRISPRa^11,13^, CRISPRi^13,14^, and CRISPRd^10^ have been individually reported in mammalian cells. However, high transformation efficiency, decent genome- editing efficiency, as well as the availability of a high throughput screening method are the prerequisites for applying MAGIC for functional genomic studies. Accompanied with the advantages of genome-scale engineering is the challenge in phenotyping large strain libraries containing millions and even billions of variants^43^. Most of current genome-scale engineering examples are limited to growth-associated phenotypes, such as substrate utilization and tolerance to toxic compounds^9,24^. Nevertheless, biosensors based on transcription factors (TFs)^44^ and responsive promoters^45^ can be integrated into the genome-scale engineering efforts. For example, a malonyl-CoA biosensor was developed and used to screen a genome-scale cDNA overexpression library^46^. In addition, robotic platforms^24,47^ and microfluidic systems^48^ are also promising solution for high throughput screening of the desired phenotypes. Overall, the combination of MAGIC and high throughput screening represents a powerful strategy to investigate fundamental biological questions, as well as engineer complex phenotypes for biotechnological applications in yeast and possibly higher eukaryotes.

## Methods

### Strains, media, and cultivation conditions

*Escherichia coli* strain NEB10β (New England Biolabs, Ipswich, MA) was used to maintain and amplify plasmids and recombinant strains were cultured at 37 °C in Luria broth medium containing 100 μg mL^−1^ ampicillin (LB/Amp). *S. cerevisiae* BY4742 was used as the host for genome-scale engineering of furfural tolerance and surface display of recombinant proteins. Yeast strains were cultivated in complex medium consisting of 2% peptone, 1% yeast extract, and 2% glucose (YPD) or synthetic complete medium consisting of 0.17% yeast nitrogen base, 0.1% mono-sodium glutamate, 0.077% CSM-URA, and 2% glucose (SED-URA) at 30 °C, 250 rpm. When necessary, 200 μg mL^−1^ G418 (KSE Scientific, Durham, NC, USA) was supplemented.

### Plasmid and strain construction

*SNR52*p-*Bsa*I-*Bsa*I-gRNA structural sequences-*SUP4*t^8^ were cloned into *Bsa*I-free pRS426 to construct gRNA expression plasmids, including p426*-LbSgH for CRISPRa, p426*-SpSgH for CRISPRi, and p426*-SaSgH for CRISPRd. Then the targeting sequences were synthesized as short oligos and cloned into the *Bsa*I sites of the helper plasmids. Yeast plasmids were isolated using a Zymoprep Yeast Plasmid Miniprep II Kit (Zymo Research, Irvine, CA) and amplified in *E. coli*. All the recombinant plasmids and oligonucleotides used in this study were listed in Supplementary Tables 9 and 10, respectively. The CRISPR-AID strain (bAID) was constructed by integrating *Pme*I-digested pAID6^8^ into the genome of BY4742 and selection for G418 resistance. The *Trichoderma reesei* endoglucanase II (EGII)-displaying strain (bAID-EG) was constructed by integrating the *TEF1p-prepro-*HisTag*-EGII-AGA1-PGK1t* cassette^8,24^ into the X4 locus of bAID. The gRNA expression cassettes identified by MAGIC screening were integrated into the predefined loci (Supplementary Table 1) in a CRISPR-assisted and marker-less manner. Recombinant yeast strains constructed in this study were listed in Supplementary Table 11.

### Design and synthesis of the MAGIC library

All ORF and RNA-coding sequences and their promoter sequences were extracted from the Saccharomyces Genome Database (SGD, https://www.yeastgenome.org). The promoter sequences, entire sequences, and coding sequences were used for the design of activation, interference, and deletion guide sequences, respectively. The desired region sequences were given to the CHOPCHOP program to generate all possible guide sequences^49,50^. All the generated guide sequences were ranked according to the binding efficiency, off-target effects, binding position, and the DNA synthesis and cloning considerations. The ranking criteria were detailed in Supplementary Table 2 and validated by the previously designed gRNAs showing high efficiency (Supplementary Table 3). For each gene, the top-six, top-six, and top-four guide sequences with the highest scores were selected for CRISPRa, CRISPRi, and CRISPRd libraries, respectively. 100 non-targeting guide sequences were included in each library as negative controls. Adapters containing priming sequences and *Bsa*I sites were added to both ends of each oligonucleotide for PCR amplification and Golden-Gate Assembly. The unique priming sequences allowed the construction of each library independently. The CRISPRa and CRISPRi oligonucleotide libraries were synthesized on a 92918-format chip, while the CRISPRd oligonucleotide library was synthesized on two 12472-format chips (CustomArray, Bothell, WA, USA) and mixed at equal molar ratio.

### Construction of the plasmid libraries

10 ng oligonucleotide pool was used as the template for PCR amplification with the corresponding primers (Supplementary Table 10). 15 ng gel-purified PCR products were assembled with 50 ng p426*-LbSgH, p426*-SpSgH, and p426*-SaSgH, respectively, using the Golden-Gate Assembly method^9,51^. The reaction mixture was transformed into NEB Turbo competent cells, yielding at least 5 × 10^6^ independent clones for each library, with ~100-fold redundancy (Table 1). Each library was plated onto 25 LB/Amp agar plates and all the bacteria were collected to extract plasmids with a Qiagen Plasmid Maxi Kit.

### Construction of the iMAGIC libraries

The yeast mutant libraries were constructed by transforming 10 μg CRISPRa, 10 μg CRISPRi, and 20 μg CRISPRd plasmid libraries, respectively, into 10 OD_600_ unit of CRISPR-AID strains using the LiAc/SS carrier DNA/PEG method^52^ with minor modification. After heat shock at 42 °C for 1 h, cells were resuspended in 4 mL YPD medium and recovered at 30 °C for ~4 h, which were then diluted 1000-fold and spread into SED-URA agar plates to evaluate the transformation efficiency. The remaining cells were cultured in 50 mL SED-URA/G418 medium for ~2 days. The independent clones for each library should be > 10^6^, with at least 30-fold redundancy. The MAGIC libraries were constructed by pooling 1 OD unit cells from each library, which would be subject to growth enrichment under stressed conditions or high throughput screening.

### iMAGIC screening of furfural tolerance

The iMAGIC libraries in triplicates were inoculated into 50 mL SED-URA/G418 medium with or without furfural in a 250 mL baffled flask. 1 OD of the mid-log phase growing cells from each of the untreated and stressed libraries were collected and the plasmids were extracted for NGS analysis. 5, 10, and 15 mM furfural were used for the first, second, and third round of iMAGIC screening, respectively. Due to the lower metabolic burdens than the plasmid-bearing strains, the integrated strains (i.e. R1, R2, and R3) were evaluated with a furfural concentration of 7.5, 12.5, and 17.5 mM, respectively. For the individually constructed strains, a single colony was pre-cultured in 2 mL SED-URA/G418 (plasmid-bearing strains) or SED/G418 (integrated strains) medium in a 14 mL round-bottom BD Falcon culture tube until saturation and then inoculated into 2 mL fresh medium supplemented with the corresponding concentration of furfural (5, 10, or 15 mM for the plasmid-harboring strains; 7.5, 12.5, or 17.5 mM for the integrated strains) with an initial OD of 0.05. Then the strains were cultivated under aerobic conditions (30 °C, 250 rpm) and furfural tolerance was evaluated by comparing the biomass accumulation when the most tolerant strain grew into mid-to-late log phase, i.e. 24, 36, and 48 h for the engineered strains constructed in the first, second, and third round of iMAGIC screening, respectively. Cell density (biomass accumulation) was determined by measuring the absorbance at 600 nm using a Tecan Infinite M1000 PRO microplate reader (Tecan Trading AG, Switzerland) and normalized to that of the control strains with an empty vector (relative biomass accumulation), unless specifically mentioned.

### iMAGIC screening of yeast surface display mutants

The iMAGIC library was cultured at 30 °C for 2 days and then subject to immunostaining and fluorescence-activated cell sorting (FACS)^8,24^. The primary and secondary antibodies were monoclonal mouse anti-histidine tag antibody (1:100 dilution, Bio-Rad, Raleigh, NC, catalog # MCA1396GA) and goat anti-mouse IgG (H + L) secondary antibody, Biotin-XX conjugate (1:100 dilution, ThermoFisher Scientific, Rockford, IL, USA, catalog # B-2763), respectively. Streptavidin, R-phycoerythrin conjugate (1:100 dilution, ThermoFisher Scientific, catalog # S866) was used to quantify the amount of biotin on the yeast surface. BD FACS Aria III cell sorting system (BD Biosciences, San Jose, CA, USA) was used for collecting the most fluorescent yeast mutants. In the first round of sorting, ~30,000 cells representing the top 1% highest fluorescence were collected. The second round sorted 96 individual yeast cells with the highest fluorescence. Then the plasmids were extracted and retransformed into the bAID-EG strain, the resulting recombinant strains were further analyzed by the cellulase activity assay. Briefly, 400 µL yeast cells were washed twice with ddH_2_O and resuspend in the same volume of 1% (w v^−1^) carboxymethyl cellulose (CMC) solution (0.1 M sodium acetate, pH 5). After incubation at 30 °C for 16 h with vigorous shaking, the amount of reducing sugars in the supernatant was quantified by a modified DNS method^8,24^. The gRNA plasmids enabling higher cellulase activity were sent for DNA sequencing.

### Construction of the sMAGIC plasmid library

100 ng of the above created plasmid libraries, LibA, LibI, and LibD, were used as the template for PCR amplification with primer sets sMAGIC-F1/sMAGIC-R1 and sMAGIC-F2/sMAGIC-R2, respectively (Supplementary Table 10). The resultant PCR products (LibA-Fg1, LibI-Fg1, and LibD-Fg1 as well as LibA-Fg2, LibI-Fg2, and LibD-Fg2) were gel purified and cloned into p426*-ccdB using Golden-Gate Assembly. The reaction mixture was transformed into NEB Turbo competent cells, yielding at least 5 × 10^7^ independent clones. Each library was plated onto 25 LB/Amp agar plates and all the bacteria were collected to extract plasmids with a Qiagen Plasmid Maxi Kit.

### Furfural-tolerance engineering using sMAGIC

The yeast mutant libraries were constructed by transforming 20 μg sMAGIC plasmid library into 10 OD_600_ unit of the bAID strain. After heat shock at 42 °C for 1 h, cells were resuspended in 4 mL YPD medium and recovered at 30 °C for ~4 h, which were then diluted 1000-fold and spread into SED-URA agar plates to evaluate the transformation efficiency. The independent clones of the sMAGIC library should be > 10^6^. The remaining cells were cultured 50 mL SED-URA/G418 medium for ~2 days and different amounts of cells (10^5^, 10^6^, and 10^7^) were spread to SED-URA/G418 agar plates containing 10 mM furfural. After incubation at 30 °C for ~3 days, many large colonies appeared and the top 96 colonies were picked and pre-cultured in 0.6 mL SED-URA/G418 medium in a deep well plate. After growth to saturation, cells were inoculated into 0.6 mL SED-URA/G418 medium with 10 mM furfural with an initial OD_600_ of 0.05 and furfural tolerance was evaluated by measuring the cell densities at 36 h. The top 16 mutants with the highest cell densities were selected and the plasmids were extracted and re-transformed into fresh bAID yeast strain to eliminate random mutagenesis. After re-transformation, the selected mutants were further verified in 2 mL SED-URA/G418 medium with 10 mM furfural in 14 mL round-bottom culture tubes and the cell densities were determined at 36 h, after inoculation. The dual-gRNA plasmids enabling the highest furfural tolerance were sent for DNA sequencing.

### Next-generation sequencing

NGS adaptors were added to the extracted plasmid libraries using the Nextera Index Kit (Illumina, San Diego, CA, USA) with a two-step PCR approach. The first step PCR added the Illumina overhang adapter sequences to all guide sequences (Supplementary Table 12) using primers AID-NGS-F1 and AID-NGS-R1. The second step PCR attached Nextera indexes to each library, and the resultant products were gel-purified and quantitated with Qubit (ThermoFisher). ~60 ng of each library was pooled, followed by quantitation by qPCR and sequencing on one lane for 161 cycles from one end of the fragments on a HiSeq 2500 using a HiSeq SBS Sequencing Kit Version 4 (Illumina).

### NGS data processing and analysis

Fastq files were generated and demultiplexed with the bcl2fastq v2.17.1.14 Conversion Software (Illumina). A bowtie index was prepared for all the designed 100,493 guide sequences and used as the reference sequences (Supplementary Data 4). From this point on, all the sequence manipulations were performed using commands on Galaxy (https://usegalaxy.org). The reads of 43 bp between *SNR52*p and *SUP4*t that contains a unique sequence in all three CRISPR-AID libraries (Supplementary Table 12) were extracted from the NGS data using FASTQ Trimmer by column (Galaxy Version 1.0.0). Extracted guide sequences were then mapped to the bowtie index using Map with Bowtie for Illumina (Galaxy Version 1.1.2) with the default settings. Unmapped reads were removed and reads mapped to each unique guide sequence were counted. The raw guide count sequence was then mapped to the original reference file and the number of reads for each guide sequences was obtained. The number of reads per guide in each library was normalized to the total read counts of that library. A threshold of one read in all six libraries (biological triplicates for untreated and furfural stressed libraries) and five-fold enrichment (normalized no. of guide in the furfural stressed library/normalized no. of guide in the untreated library) for each replicate was set to keep a guide sequence. The targets with the highest average folds of enrichment were chosen for further verification.

### Quantitative PCR analysis

Mid-log-phase yeast cells were collected to extract total RNAs using the RNeasy Mini Kit (QIAGEN, Valencia, CA, USA) following the manufacturer’s instructions. 2 µg of the RNA samples were then reversed transcribed into cDNA using the Transcriptor First Strand cDNA Synthesis Kit using oligo-dT primer (Roche, Indianapolis, IN, USA). The qPCR experiments were carried out using SYBR Green-based method using the Roche LightCycler 480 System.

### Fermentation and HPLC analysis

A single colony of WT and R3 were inoculated into 3 mL SED/G418 medium and cultured until saturation, which was then transferred into 50 mL fresh SED/G418 medium with or without the supplementation of 17.5 mM furfural in a 250 mL un-baffled shaker flask with an initial OD of 0.05. Fermentation was performed under oxygen-limited conditions (30 °C and 100 rpm), and samples were taken every 24 h and analyzed by HPLC. Glucose and ethanol were quantified using a Shimadzu HPLC (Columbia, MD, USA) equipped with an Aminex HPX-87H column (Bio-Rad) and Shimadzu RID-10A refractive index detector. The column was kept at 65 °C with 0.5 mM sulfuric acid solution at a flow rate of 0.6 mL min^−1^ as the mobile phase. Furfural and furfuryl alcohol were quantified using HPLC^25^ with an Agilent ZORBAX 80A Extend-C18 column (Agilent Technologies, Palo Alto, CA, USA) and a Shimadzu SPD-20A UV–Vis Detector (277 nm for furfural and 210 nm for furfuryl alcohol). The mobile phase was acetonitrile/water solution, 5% for 15 min, 100% for 5 min, and then 5% for 5 min, with a flow rate of 1.0 mL min^−1^.

### Reporting summary

Further information on research design is available in the Nature Research Reporting Summary linked to this article.

## Supplementary information

Supplementary Information

Description of Additional Supplementary Files

Supplementary Data 1

Supplementary Data 2

Supplementary Data 3

Supplementary Data 4

Reporting Summary

## Data Availability

All relevant data are available from the authors upon reasonable request. The raw reads of the NGS data were deposited into the NCBI Sequence Read Archive (SRA) database (accession number: PRJNA504483). Plasmids constructed in this study are available from Addgene [https://www.addgene.org/browse/article/28207401/]. The source data underlying Figs. 2b, d, f, 3a–f, 4, and 5b, d and Supplementary Figs. 1, 4, 6, 7a, c, 9 and 10 are provided as a Source Data file.

## Source data

Source Data
