# Supplementary material for: Multi-functional genome-wide CRISPR system for high throughput genotype–phenotype mapping
Source: Nat Commun. 2019 Dec 19;10:5794. doi: 10.1038/s41467-019-13621-4 (PMC6923430; doi:10.1038/s41467-019-13621-4)
Supplement: Supplementary file 1 — Supplementary Information [file 41467_2019_13621_MOESM1_ESM.docx]

**Multi-functional Genome-wide CRISPR System for High Throughput Genotype-Phenotype Mapping**

Lian *et al.*


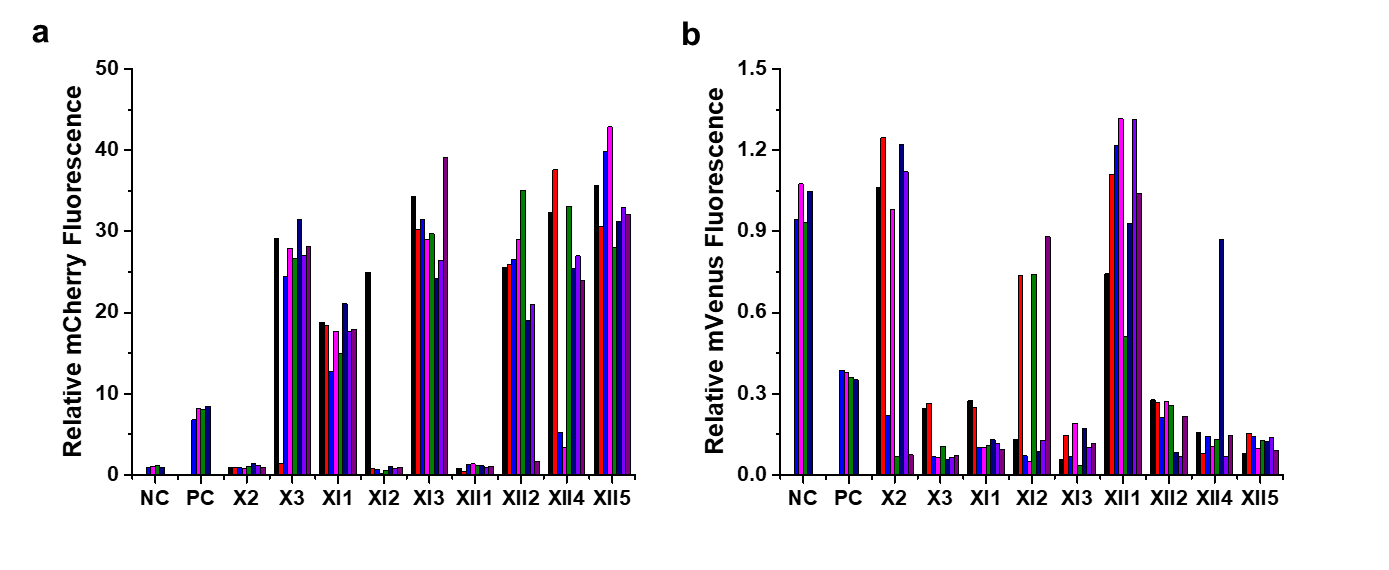


**Supplementary Figure 1.** Characterization of the integration and gRNA expression efficiency of the pre-selected genomic loci. The integration efficiency and gRNA expression level were evaluated by co-transforming the reporter strain (bAID-RV) with gRNA plasmid as well as its corresponding linear donor fragment, which contained a gRNA expression cassette to activate the expression of mCherry or to repress the expression of mVenus. Eight colonies were randomly picked up to measure the change in fluorescence intensities. The mVenus and mCherry fluorescence signals were measured at 514–528 nm and 587–610 nm, respectively, using a Tecan Infinite M1000 PRO multimode reader (Tecan Trading AG, Switzerland). The fluorescence intensity (relative fluorescence units; RFU) was normalized to cell density that was determined by measuring the absorbance at 600 nm using the same microplate reader. NC indicates the absence of any targeting gRNA; PC for CRISPRa includes a gRNA expression plasmid for mCherry activation, while PC for CRISPRi includes a gRNA expression plasmid for mVenus repression. The higher activation or repression efficiency of the integrated gRNA than its plasmid counterpart might result from lower metabolic burdens. The source data are provided as a Source Data file.

**
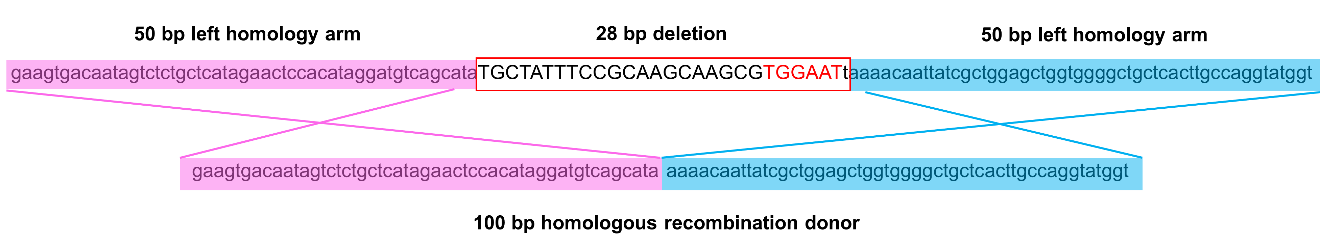
**

**Supplementary Figure 2.** Design of gRNA for genome-scale CRISPRd library. The HI-CRISPR[^1^](#_ENREF_1) design was adopted to combine the homology donor (100 bp) and targeting sequences (21 bp) in the same vector. The guide sequence is capitalized and the PAM sequence is shown in red. To ensure the disruption of the target gene, a 28-bp region (boxed) is deleted, including the 21 bp guide sequence, 6 bp PAM sequence, and one extra nucleotide following the PAM sequence. The left and right homology arms are shown in pink and blue, respectively. The design of gRNA for the deletion of *ADE2* was shown as an example.

**
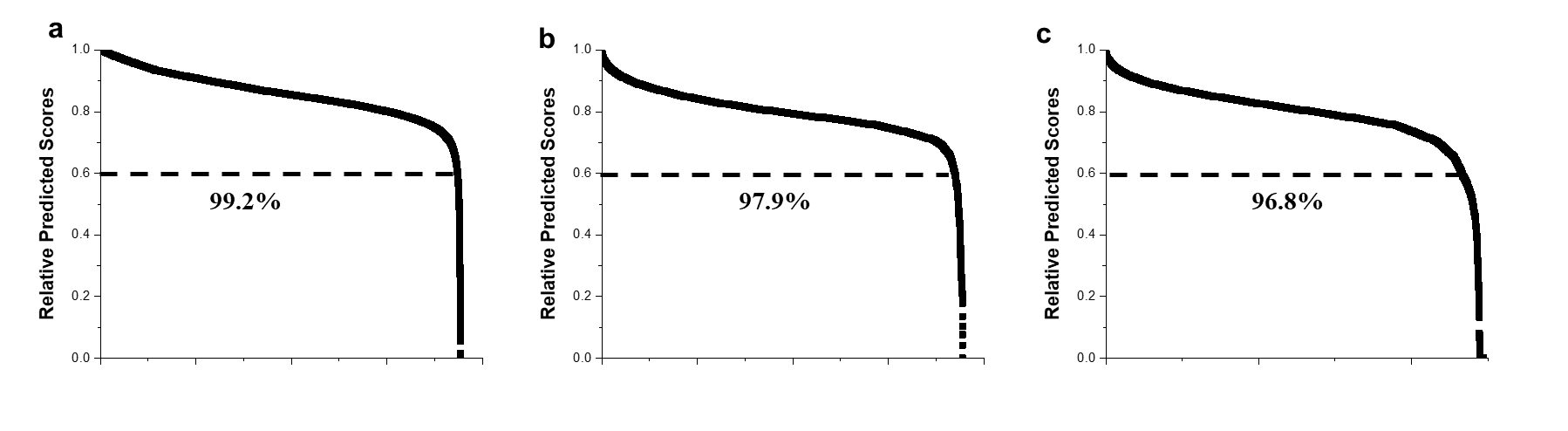
**

**Supplementary Figure 3.** Score distribution of the designed guide sequences for the genome-scale activation (**a**), and interference (**b**), and deletion (**c**) libraries, respectively. Based on the score equation detailed in **Supplementary Table 2**, the highest score for activation, interference, and deletion libraries are 3, 4, and 4, respectively. The dashed line represents the percentage of gRNAs with high scores (higher than 60% of the maximal score).


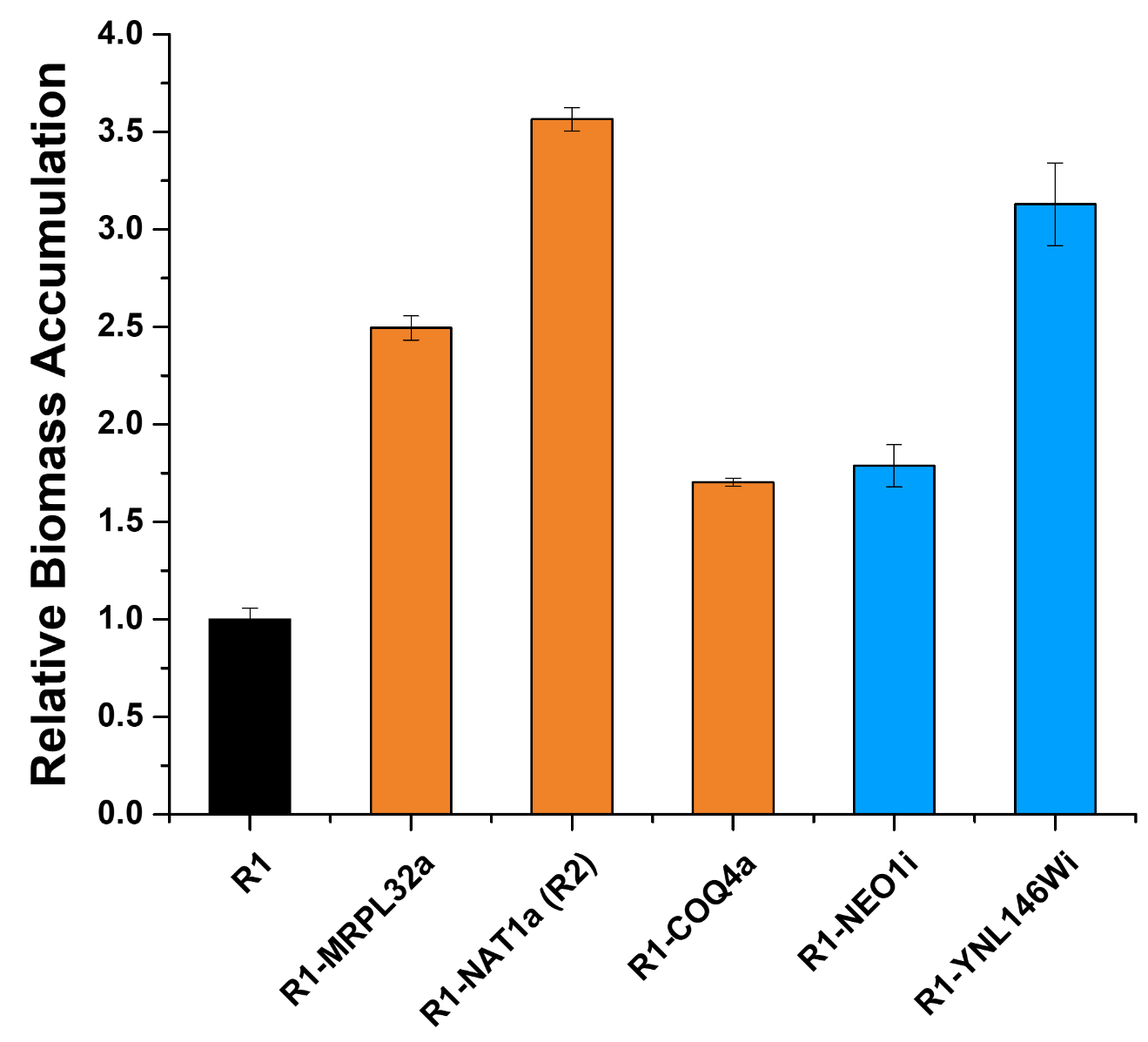


**Supplementary Figure 4.** Verification of the second round iMAGIC screening identified targets when integrated into the X4 locus of R1 strain (*SIZ1i*). The strains were pre-cultured in SED medium until saturation and then inoculated into fresh SED medium supplemented with 12.5 mM furfural. The cell density was measured in 36 h. Error bars represent the mean ± s.d. of biological triplicates. The source data are provided as a Source Data file.

**
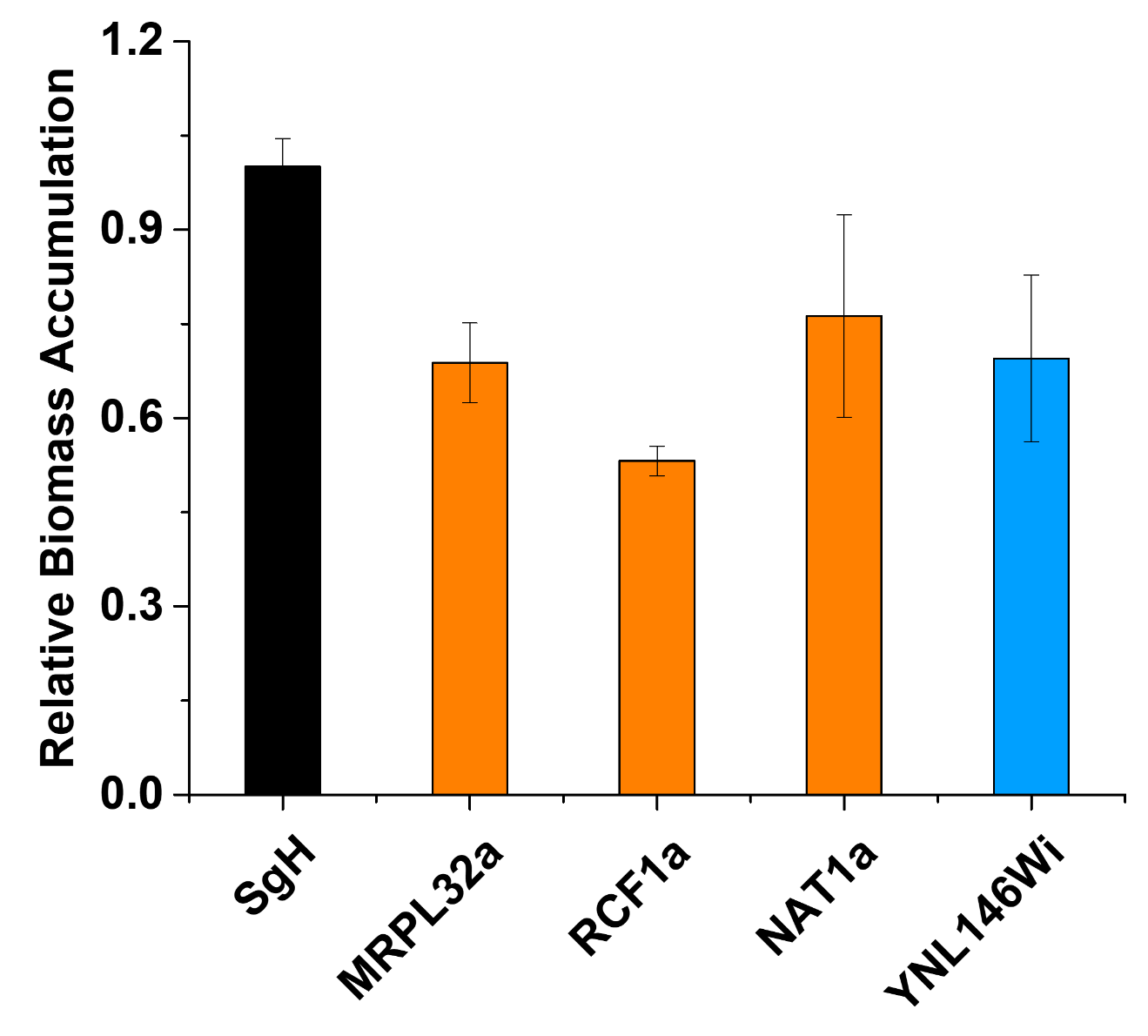
**

**Supplementary Figure 5.** Dependence of the second round iMAGIC identified targets on *SIZ1i*. The second round iMAGIC identified targets (*YNL146Wi*, *MRPL32a*, *RCF1a*, and *NAT1a*) were introduced into the wild-type strain and the furfural tolerance was compared with the negative control strain (SgH). The plasmid borne strains were pre-cultured in SED-URA/G418 medium until saturation and then inoculated into fresh SED-URA/G418 medium supplemented with 10 mM furfural. The cell density was measured in 36 h. Error bars represent the mean ± s.d. of biological triplicates. The source data are provided as a Source Data file.


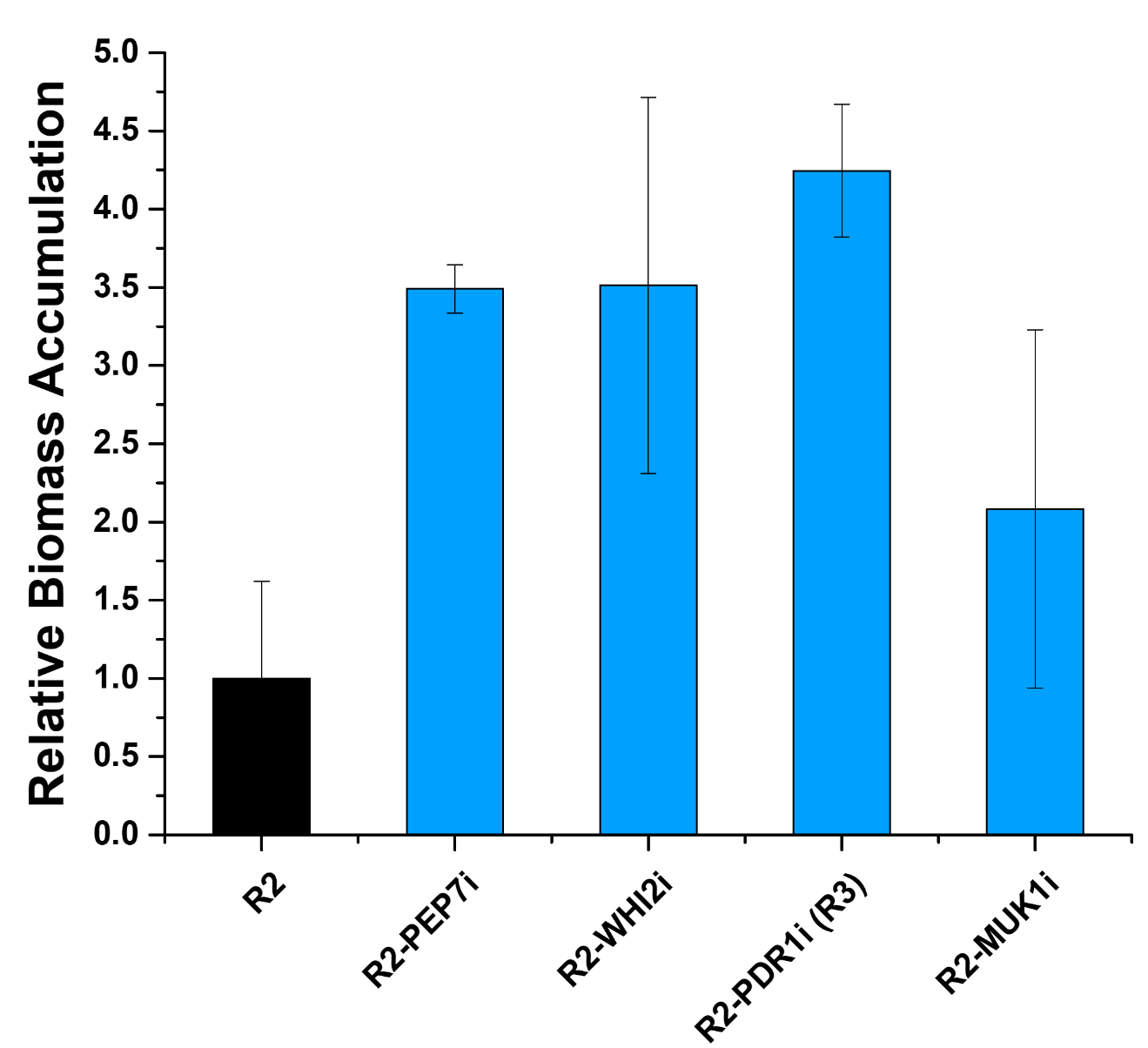


**Supplementary Figure 6.** Verification of the third round iMAGIC screening identified targets when integrated into the XI1 locus of the R2 strain (*SIZ1i*-*NAT1a*). The strains were pre-cultured in SED medium until saturation and then inoculated into fresh SED medium supplemented with 17.5 mM furfural. The cell density was measured in 48 h. Error bars represent the mean ± s.d. of biological triplicates. The source data are provided as a Source Data file.

**
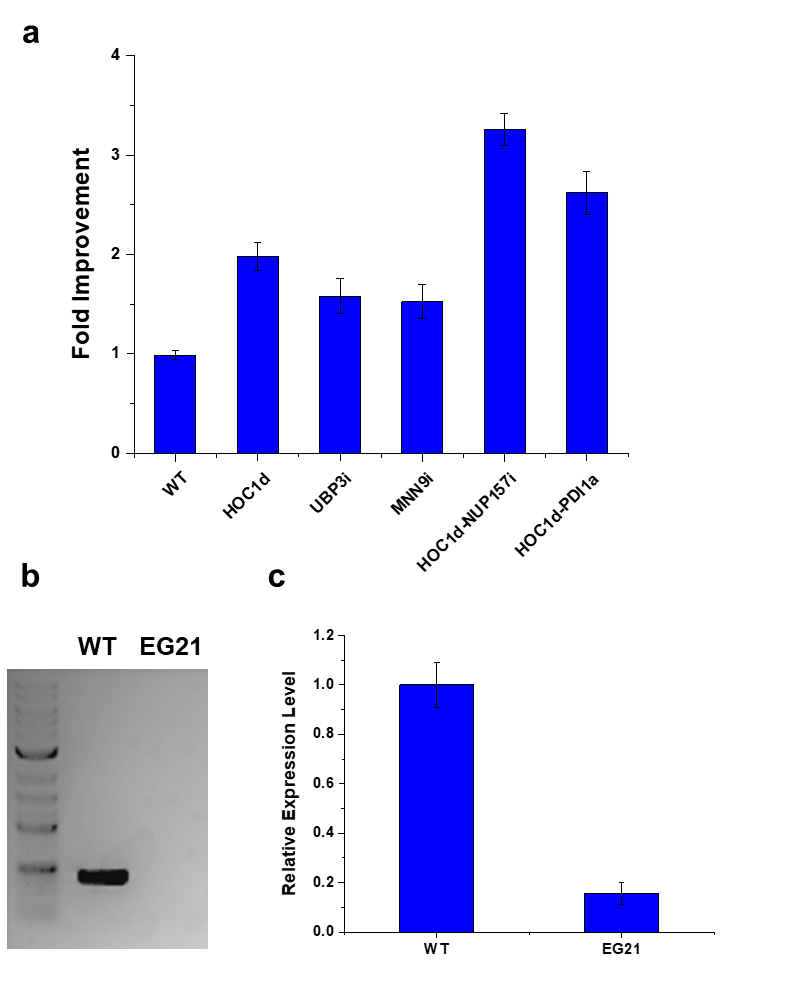
**

**Supplementary Figure 7.** Identification of genetic determinants of yeast surface display of recombinant proteins by iMAGIC. (**a**) The 1^st^ round of iMAGIC screening identified *HOC1d* as the best target, followed by *UBP3i* and *MNN9i*. The 2^nd^ round of iMAGIC screening identified *NUP157i* and *PDI1a* as the top candidates that worked synergistically with *HOC1d* to improve display levels of recombinant proteins on yeast surface. The cellulase activity of WT (bAID-EG), EG11 (*HOC1d*), EG12 (*UBP3i*), EG13 (*MNN9i*), EG21 (*HOC1d-NUP157i*), and EG22 (*HOC1d-PDI1a*) were measured and compared. The deletion of *HOC1* and interference of *NUP157* in EG21 were confirmed by diagnostic PCR (**b**) and qPCR (**c**), respectively. Error bars represent the mean ± s.d. of biological triplicates. The source data for figures **a** and **c** are provided as a Source Data file.

**
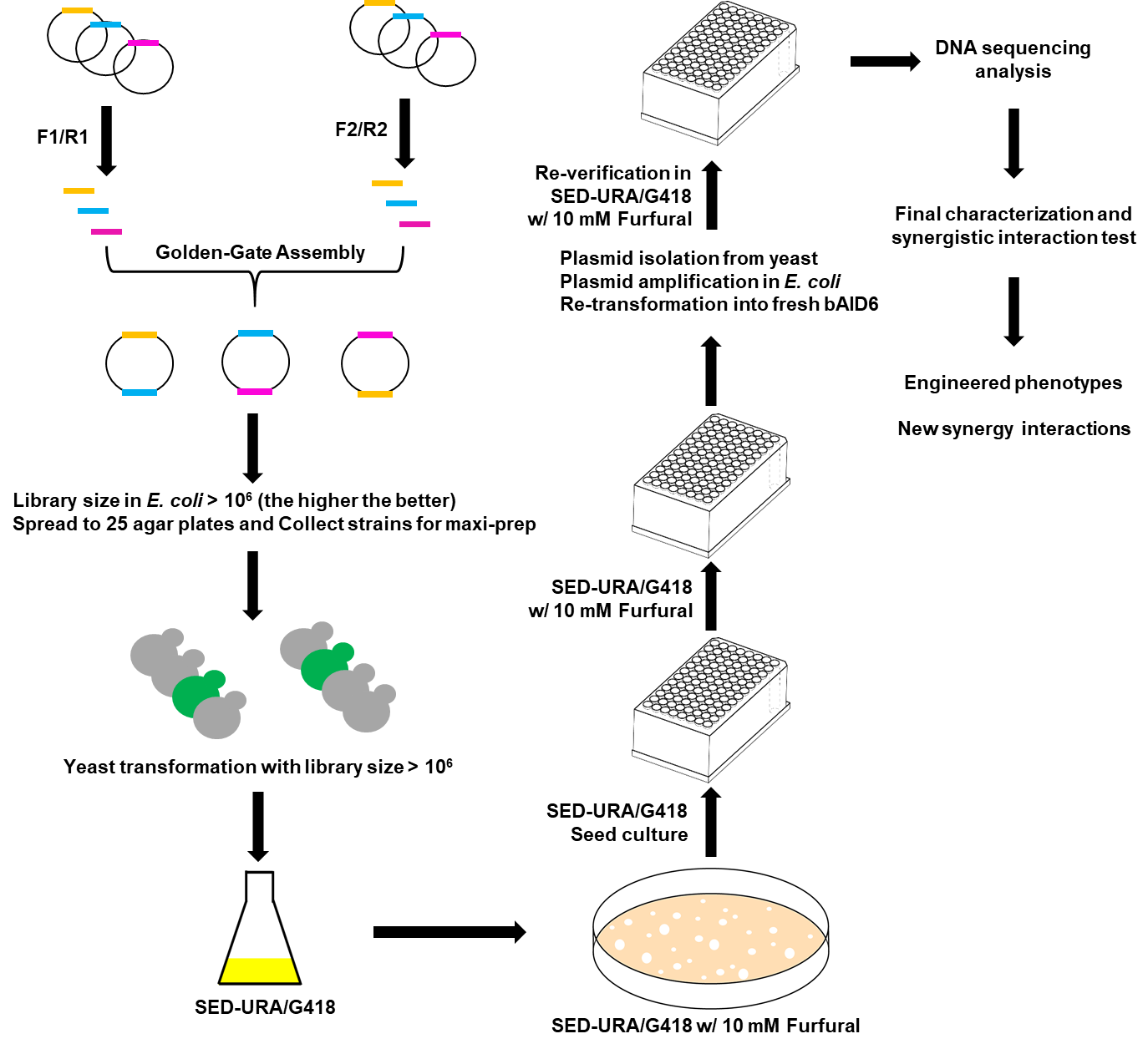
**

**Supplementary Figure 8.** A brief overview of the construction of the sMAGIC library and its application in engineering furfural tolerance. sMAGIC can not only engineer complex phenotypes, but also identify synergistic interactions among gain-, reduction-, and loss-of-function targets beyond the reach of iMAGIC.

**Supplementary Figure 9.** Comparison of the furfural tolerance of the engineered yeast strains obtained by two rounds of iMAGIC and CHAnGE screening. The WT, R1 (*SIZ1i*), R2 (*SIZ1i-NAT1a*) and CHAnGE strain (*SIZ1d-LCB3d*)[^1^](#_ENREF_1) were pre-cultured in SED until saturation and then inoculated into fresh SED medium supplemented with 10 mM furfural with an initial OD of 0.05. Error bars represent the mean ± s.d. of biological triplicates. The source data are provided as a Source Data file.

**Supplementary Figure 10.** Comparison of the furfural tolerance of the iMAGIC engineered yeast strain R3 with that obtained using rational metabolic engineering. The highest furfural tolerance with clear genetic background was achieved by the overexpression *TPS1* and *ARI1* as well as the disruption of *NTH1*[^2^](#_ENREF_2). The previously reported strain was reconstructed by integrating the *TPS1* overexpression cassette into the *NTH1* locus and *ARI1* overexpression cassette into the X4 locus of the bAID strain (*TPS1a-ARI1a-NTH1d*). The WT, R3 (*SIZ1i-NAT1a-PDR1i*) and the metabolic engineering strain (*TPS1a-ARI1a-NTH1d*) were pre-cultured in YPD until saturation and then inoculated into YPD medium supplemented with 30 mM furfural with an initial OD of 0.1. Error bars represent the mean ± s.d. of biological duplicates. The source data are provided as a Source Data file.

**Supplementary Table 1.** Characterization of the genomic loci for marker-less integration of gRNA expression cassettes. The gRNA targeting efficiency was tested by transforming the gRNA plasmid without any donor to repair the double strand break, and efficient gRNA should result in no survived colonies. The integration efficiency and gRNA expression level were evaluated by co-transforming the reporter strain (bAID-RV) with gRNA plasmid as well as its corresponding linear donor fragment, which contained a gRNA expression cassette to activate the expression of mCherry or to repress the expression of mVenus. Eight colonies were randomly picked to measure the change in fluorescence intensities. The corresponding results were shown in **Supplementary Fig. 1**. The loci and the corresponding gRNAs chosen for CRISPR-assisted and marker-less genome integration were shown in blue.

| Site | A | I | Sum of A and I | No donor | gRNA |
| --- | --- | --- | --- | --- | --- |
| X2 | 0/8 | 3/8 | 3/16 | Confluent | pSg334 |
| X3 | 7/8 | 8/8 | 15/16 | 4 | pSg335 |
| X4 |  |  |  | 0 | pSg336 |
| XI1 | 8/8 | 8/8 | 16/16 | 30 | pSg337 |
| XI2 | 1/8 | 5/8 | 6/16 | Confluent | pSg338 |
| XI3 | 8/8 | 8/8 | 16/16 | 1 | pSg339 |
| XII1 | 0/8 | 0/8 | 0/16 | Confluent | pSg340 |
| XII2 | 7/8 | 8/8 | 15/16 | 20 | pSg341 |
| XII4 | 6/8 | 7/8 | 13/16 | 20 | pSg342 |
| XII5 | 8/8 | 8/8 | 16/16 | 1 | pSg343 |

**Supplementary Table 2.** Criteria for scoring of the guide sequences for the CRISPRa, CRISPRi, and CRISPRd libraries.

|  | LibA | LibI | LibD |
| --- | --- | --- | --- |
| Efficiency score E^1^ | 0 | CHOPCHOP | CHOPCHOP |
| Position score a^2^ | a=\|X-250\|/250 | if X<0, a=\|X+125\|/125;  if X>=0 and from T a=0.25  if X>=0 and from NT a=1 | if X/CDS<1/3 a=0  if 1/3=<X/CDS<=2/3 a=0.2  if X/CDS>2/3 a=0.5 |
| GC score b | if 40-60% b=0  if 30-40% or 60-70% b=0.2  if 20-30% or 70-80% b=0.4  if 10-20% or 80-90% b=0.6  if 0-10% or 90-100% b=0.8 | | |
| Off-target score c^3^ | c=(SM+MM0+MM1+MM2+MM3)/20 | | |
| PolyT score d^4^ | if ConsecutiveT<4 d=0  if ConsecutiveT>4 d= ConsecutiveT/10 | | |
| PolyG score e^5^ | if consecutiveG>5 e=0  else e=1 | | |
| BsaI score f^6^ | if BsaI f=0  else f=1 | | |
| Diversity score g^7^ | if distance<10bp g=0  else g=1 | | |
| Total Score S | S=(3+E-a-b-c-d)*e*f*g | | |

Notes:

^1^Efficiency score is from COPCHOP[^3^](#_ENREF_3), and the computational program for the efficiency score of Cpf1 was not available when the library sequences were designed. Therefore, the highest scores for the activation, interference, and deletion gRNA libraries are 3, 4, and 4, respectively.

^2^X represents the gRNA binding site, with X=0 presenting the start codon (ATG). Based on previous experience, CRIAPRa is the most active when binding to ~200 bp upstream of the transcription starting site (TSS) or ~250 bp upstream of the start codon); the efficiency of CRISPRi is the highest when targeting to the promoter region (~75 bp upstream of TSS or ~125 bp upstream of the start codon) and the template strand (T) of the coding sequences; for gene disruption, it is better to target the 5’-end of the coding sequences.

^3^SC and MM scores are from CHOPCHOP. SC, self-complementarity; MM0, no mismatch; MM1, 1 mismatch; MM2, 2 mismatches; MM3, 3 mismatches.

^4^PolyT may be read as a terminator by the Type III RNA polymerase.

^5^PolyG is difficult for DNA synthesis.

^6^BsaI is used for the cloning of the gRNA plasmid libraries.

^7^The gRNAs cluster together may have similar targeting efficiency and it may result in low library diversity.

**Supplementary Table 3.** Validation of the gRNA ranking criteria. Most of the previously designed gRNAs[^4^](#_ENREF_4) with high efficiency was found to be highly ranked in the designed genome-scale CRISPRa, CRISPRi, and CRISPRd libraries.

| CRISPRa | gRNA^1^ | Ranking | CRISPRi | gRNA | Ranking | CRISPRd | gRNA | Ranking |
| --- | --- | --- | --- | --- | --- | --- | --- | --- |
| *CCW12* | Sg217 | 4 | *CYS4* | Sg246 | 3 | *ADE2* | Sg93 | 3 |
| *ERO1* | Sg218 | Close to 1 |  | Sg247 | 11 |  | Sg94 | 5 |
| *GAL11* | Sg242 | 3 |  | Sg248 | Close to 5 |  | Sg95 | 10 |
|  | Sg243 | 5 | *ERG9* | Sg170 | Close to 1 | *PEP1* | Sg265 | 2 |
| *HMG1* | Sg175 | 1 |  | Sg171 | Close to 2 | *ADO1* | Sg255 | 2 |
|  | Sg176 | 3 |  | Sg172 | 3 | *ROX1* | Sg186 | 7 |
|  | Sg177 | 6 |  | Sg173 | 4 | *VPS8* | Sg266 | 1 |
| *MET6* | Sg252 | 1 |  | Sg174 | 12 | *YPS1* | Sg267 | 2 |
|  | Sg253 | 2 | *KEX2* | Sg262 | 9 |  |  |  |
|  | Sg254 | 3 |  | Sg263 | 3 |  |  |  |
| *PEX5* | Sg194 | 2 |  | Sg264 | 1 |  |  |  |
|  | Sg195 | Close to 4 | *MNN9* | Sg230 | 4 |  |  |  |
| *PTI1* | Sg196 | Close to 3 |  | Sg231 | Close to 5 |  |  |  |
|  | Sg197 | 5 | *OCH1* | Sg227 | 1 |  |  |  |
| *SAM2* | Sg244 | Close to 1 |  | Sg228 | 9 |  |  |  |
|  | Sg245 | 3 |  | Sg229 | 4 |  |  |  |
| *SEC1* | Sg222 | 2 | *PMR1* | Sg204 | 2 |  |  |  |
| *SSO1* | Sg224 | 1 |  | Sg260 | 6 |  |  |  |
|  |  |  |  | Sg261 | Close to 3 |  |  |  |
|  |  |  | *SED1* | Sg198 | 1 |  |  |  |
|  |  |  |  | Sg199 | 3 |  |  |  |
|  |  |  |  | Sg200 | 2 |  |  |  |
|  |  |  | *YCH1* | Sg201 | Close to 2 |  |  |  |
|  |  |  |  | Sg202 | 1 |  |  |  |
|  |  |  |  | Sg203 | 4 |  |  |  |
|  |  |  | *TEF1* | Sg28 | 3 |  |  |  |
|  |  |  |  | Sg27 | 10 |  |  |  |

**Supplementary Table 4.** Design of oligonucleotides for CRISPRa, CRISPRi, and CRISPRd libraries. The priming sites are underlined, *Bsa*I sites for golden-gate assembly are highlighted in yellow, guide sequences are shown in green, and the homology donor for HI-CRISPR[^1^](#_ENREF_1) gene deletion is shown in blue.

|  | Sequences (5’ to 3’) |
| --- | --- |
| LibA | TCCTTAAGTGGTCCGTGTTCGGACCTAATCGGTCTCcagatNNNNNNNNNNNNNNNNNNNNNNNttttcGAGACCACGTCGTCCATTCTTAGAGGTGGCAGCTGG |
| LibI | CGGAGCAGACATTGTAAGGCTACGTTCACCGGTCTCcgatcNNNNNNNNNNNNNNNNNNNNgtttcGAGACCCGTCCAACGAAGATAGCACGAGAGGCCTAC |
| LibD | GTATCTCGCAGCCGGTCTCcgatcNNNNNNNNNNNNNNNNNNNNNNNNNNNNNNNNNNNNNNNNNNNNNNNNNNNNNNNNNNNNNNNNNNNNNNNNNNNNNNNNNNNNNNNNNNNNNNNNNNNNNNNNNNNNNNNNNNNNNNNNNgtttcGAGACCACGAGAGAGAACCG |

**Supplementary Table 5.** Guide sequence distribution of the designed CRISPRa, CRISPRi, and CRISPRd libraries. Notably, 100 randomly generated guide sequences in each library were not included in this table.

|  | Genes Targeting | | | | | | Total Genes | Total Guides |
| --- | --- | --- | --- | --- | --- | --- | --- | --- |
| No. of guides | 1 | 2 | 3 | 4 | 5 | 6 |  |  |
| No. of genes in LibA | 0 | 1 | 6 | 10 | 11 | 6267 | 6295 | 37717 |
| No. of genes in LibI | 0 | 0 | 0 | 0 | 0 | 6295 | 6295 | 37770 |
| No. of genes in LibD | 44 | 111 | 108 | 6029 | 0 | 0 | 6295 | 24706 |

**Supplementary Table 6.** Genotyping of the diversity of the iMAGIC library, with the CRISPRa yeast library shown as an example. 50 colonies were randomly picked from the CRISPRa yeast library (LibA) and cultured in 2 mL SED-URA/G418 medium until saturation. Then plasmids were extracted from the yeast cells using a Zymoprep Yeast Plasmid Miniprep II Kit and retransformed into and amplified in *E. coli* for DNA sequencing. Notably, 6 out of 50 samples resulted in sequencing failure and were not included for diversity analysis.

| No. | Target | No. | Target | No. | Target |
| --- | --- | --- | --- | --- | --- |
| 1 | *SMC3* | 16 | *SET3* | 31 | *UBP3* |
| 2 | *ASG1* | 17 | *APT2* | 32 | *VPS30* |
| 3 | *RXT3* | 18 | *2693* | 33 | *SNR68* |
| 4 | *PDE1* | 19 | *LPD1* | 34 | *PRM2* |
| 5 | *RAD33* | 20 | *COX7* | 35 | *ERG9* |
| 6 | *ADE3* | 21 | *SKP1* | 36 | *RPS12* |
| 7 | *FYV4* | 22 | *NUP170* | 37 | *SNR44* |
| 8 | *ERG29* | 23 | *DED81* | 38 | *2682* |
| 9 | *BRE2* | 24 | *EXG1* | 39 | *ADE57* |
| 10 | *RXT3* | 25 | *RKM5* | 40 | *SDS22* |
| 11 | *SWP1* | 26 | *RSM22* | 41 | *CAF40* |
| 12 | *SNF12* | 27 | *MAK21* | 42 | *HMG1* |
| 13 | *4813* | 28 | *AYR1* | 43 | *1478* |
| 14 | *SEC65* | 29 | *GIM3* | 44 | *APC2* |
| 15 | *SPA2* | 30 | *DED81* |  |  |

**Supplementary Table 7.** Functional annotation of the MAGIC screening identified genetic targets from SGD (Saccharomyces Genome Database, https://www.yeastgenome.org).

|  | AID | Function |
| --- | --- | --- |
| *SPC97* | A | Component of the microtubule-nucleating Tub4p (gamma-tubulin) complex; interacts with Spc110p at the spindle pole body (SPB) inner plaque and with Spc72p at the SPB outer plaque |
| *BUD22* | A | Protein required for rRNA maturation and ribosomal subunit biogenesis; required for 18S rRNA maturation; also required for small ribosomal subunit biogenesis; cosediments with pre-ribosomal particles; mutation decreases efficiency of +1 Ty1 frameshifting and transposition, and affects budding pattern |
| *SIZ1* | I | SUMO E3 ligase; promotes attachment of small ubiquitin-related modifier sumo (Smt3p) to primarily cytoplasmic proteins; regulates Rsp5p ubiquitin ligase activity and is in turn itself regulated by Rsp5p; required for sumoylation of septins and histone H3 variant Cse4p, a prerequisite for STUbL-mediated Ub-dependent degradation; localizes to the septin ring; acts as an adapter between E2, Ubc9p and substrates; tends to compensate for survival of DNA damage in absence of Nfi1p |
| *SLX5* | I | Subunit of the Slx5-Slx8 SUMO-targeted Ub ligase (STUbL) complex; role in Ub-mediated degradation of histone variant Cse4p preventing mislocalization to euchromatin; role in proteolysis of spindle positioning protein Kar9p, and DNA repair proteins Rad52p and Rad57p; forms SUMO-dependent nuclear foci, including DNA repair centers; contains a RING domain and two SIM motifs; associates with the centromere; required for maintenance of genome integrity like human ortholog RNF4 |
| *NUP133* | I | Subunit of Nup84p subcomplex of nuclear pore complex (NPC); contributes to nucleocytoplasmic transport, NPC biogenesis; is involved in establishment of a normal nucleocytoplasmic concentration gradient of GTPase Gsp1p; also plays roles in several processes that may require localization of genes or chromosomes at nuclear periphery, including double-strand break repair, transcription and chromatin silencing; relocalizes to cytosol in response to hypoxia; homolog of human NUP133 |
| *GPI17* | I | Transmembrane protein; subunit of the glycosylphosphatidylinositol transamidase complex that adds GPIs to newly synthesized proteins; human PIG-S homolog |
| *UME1* | I | Component of both the Rpd3S and Rpd3L histone deacetylase complexes; negative regulator of meiosis; required for repression of a subset of meiotic genes during vegetative growth, binding of histone deacetylase Rpd3p required for activity, contains a NEE box and a WD repeat motif; homologous with Wtm1p; UME1 has a paralog, WTM2, that arose from the whole genome duplication |
| *SAP30* | D | Component of Rpd3L histone deacetylase complex; involved in silencing at telomeres, rDNA, and silent mating-type loci; involved in telomere maintenance |
| *MRPL32* | A | Mitochondrial ribosomal protein of the large subunit; protein abundance increases in response to DNA replication stress |
| *ASE1* | A | Mitotic spindle midzone-localized microtubule bundling protein; microtubule-associated protein (MAP) family member; required for spindle elongation and stabilization; undergoes cell cycle-regulated degradation by anaphase promoting complex; potential Cdc28p substrate; relative distribution to microtubules decreases upon DNA replication stress |
| *RCF1* | A | Cytochrome c oxidase subunit; required for assembly of the Complex III-Complex IV supercomplex, and for assembly of Cox13p and Rcf2p into cytochrome c oxidase; similar to Rcf2p, and either Rcf1p or Rcf2p is required for late-stage assembly of the Cox12p and Cox13p subunits and for cytochrome c oxidase activity; required for growth under hypoxic conditions; member of the hypoxia induced gene family; C. elegans and human orthologs are functional in yeast |
| *NAT1* | A | Subunit of protein N-terminal acetyltransferase NatA; NatA comprised of Nat1p, Ard1p, and Nat5p; N-terminally acetylates many proteins to influence multiple processes such as cell cycle progression, heat-shock resistance, mating, sporulation, telomeric silencing and early stages of mitophagy; orthologous to human NAA15; expression of both human NAA10 and NAA15 functionally complements ard1 nat1 double mutant although single mutations are not complemented by their orthologs |
| *NRT1* | A | High-affinity nicotinamide riboside transporter; also transports thiamine with low affinity; major transporter for 5-aminoimidazole-4-carboxamide-1-beta-D-ribofuranoside (acadesine) uptake; shares sequence similarity with Thi7p and Thi72p; proposed to be involved in 5-fluorocytosine sensitivity |
| *COQ4* | A | Protein with a role in ubiquinone (Coenzyme Q) biosynthesis; possibly functioning in stabilization of Coq7p; located on matrix face of mitochondrial inner membrane; component of a mitochondrial ubiquinone-synthesizing complex; human homolog COQ4 can complement yeast coq4 null mutant |
| *NEO1* | I | Phospholipid translocase (flippase), role in phospholipid asymmetry of plasma membrane; involved in endocytosis, vacuolar biogenesis and Golgi to ER vesicle-mediated transport; localizes to endosomes and the Golgi apparatus |
| *YNL146W* | I | Putative protein of unknown function; green fluorescent protein (GFP)-fusion protein localizes to the endoplasmic reticulum; YNL146W is not an essential gene |
| *tH(GUG)K* | I | Histidine tRNA (tRNA-His) |
| *SNU66* | I | Component of the U4/U6.U5 snRNP complex; involved in pre-mRNA splicing via spliceosome; also required for pre-5S rRNA processing and may act in concert with Rnh70p; has homology to human SART-1 |
| *DDL1* | I | DDHD domain-containing phospholipase A1; mitochondrial matrix enzyme with sn-1-specific activity, hydrolyzing cardiolipin, PE, PC, PG and PA; implicated in remodeling of mitochondrial phospholipids; antagonistically regulated by Aft1p and Aft2p; in humans, mutations in DDHD1 and DDHD2 genes cause specific types of hereditary spastic paraplegia, while DDL1-defective yeast share similar phenotypes such as mitochondrial dysfunction and defects in lipid metabolism |
| *ECM31* | D | Ketopantoate hydroxymethyltransferase; required for pantothenic acid biosynthesis, converts 2-oxoisovalerate into 2-dehydropantoate |
| *YNR064C* | A | Epoxide hydrolase; member of the alpha/beta hydrolase fold family; may have a role in detoxification of epoxides |
| *MGR1* | A | Subunit of the mitochondrial (mt) i-AAA protease supercomplex; i-AAA degrades misfolded mitochondrial proteins; forms a subcomplex with Mgr3p that binds to substrates to facilitate proteolysis; required for growth of cells lacking mtDNA |
| *PEP7* | I | Adaptor protein involved in vesicle-mediated vacuolar protein sorting; multivalent adaptor protein; facilitates vesicle-mediated vacuolar protein sorting by ensuring high-fidelity vesicle docking and fusion, which are essential for targeting of vesicles to the endosome; required for vacuole inheritance |
| *VPS8* | I | Membrane-binding component of the CORVET complex; involved in endosomal vesicle tethering and fusion in the endosome to vacuole protein targeting pathway; interacts with Vps21p; contains RING finger motif |
| *ZRT1* | I | High-affinity zinc transporter of the plasma membrane; responsible for the majority of zinc uptake; transcription is induced under low-zinc conditions by the Zap1p transcription factor |
| *WHI2* | I | Protein required for full activation of the general stress response; required with binding partner Psr1p, possibly through Msn2p dephosphorylation; regulates growth during the diauxic shift; negative regulator of G1 cyclin expression; SWAT-GFP, seamless-GFP and mCherry fusion proteins localize to the cell periphery |
| *PDR1* | I | Transcription factor that regulates the pleiotropic drug response; zinc cluster protein that is a master regulator involved in recruiting other zinc cluster proteins to pleiotropic drug response elements (PDREs) to fine tune the regulation of multidrug resistance genes; relocalizes to the cytosol in response to hypoxia; PDR1 has a paralog, PDR3, that arose from the whole genome duplication |
| *MUK1* | I | Guanine nucleotide exchange factor (GEF); involved in vesicle-mediated vacuolar transport, including Golgi-endosome trafficking and sorting through the multivesicular body (MVB); specifically stimulates the intrinsic guanine nucleotide exchange activity of Rab family members (Vps21p/Ypt52p/Ypt53p); partially redundant with GEF VPS9; required for localization of the CORVET complex to endosomes; contains a VPS9 domain |
| *NHP10* | D | Non-essential INO80 chromatin remodeling complex subunit; preferentially binds DNA ends, protecting them from exonucleatic cleavage; deletion affects telomere maintenance via recombination; related to mammalian high mobility group proteins |
| *SFH1* | A | Component of the RSC chromatin remodeling complex; essential gene required for cell cycle progression and maintenance of proper ploidy; phosphorylated in the G1 phase of the cell cycle; Snf5p paralog; hSNF5 tumor suppressor ortholog |
| *UBC9* | I | SUMO-conjugating enzyme involved in the Smt3p conjugation pathway; nuclear protein required for S- and M-phase cyclin degradation and mitotic control; involved in proteolysis mediated by the anaphase-promoting complex cyclosome (APCC) |
| *SDS3* | I | Component of the Rpd3L histone deacetylase complex; required for its structural integrity and catalytic activity, involved in transcriptional silencing and required for sporulation; relocalizes to the cytosol in response to hypoxia; cells defective in SDS3 display pleiotropic phenotypes |
| *SPC29* | I | Inner plaque spindle pole body (SPB) component; links the central plaque component Spc42p to the inner plaque component Spc110p; required for SPB duplication |

**Supplementary Table 8.** Genotyping of the diversity of the sMAGIC library. 40 colonies were randomly picked from the yeast library and cultured in 2 mL SED-URA/G418 medium until saturation. Then plasmids were extracted from the yeast cells using a Zymoprep Yeast Plasmid Miniprep II Kit and retransformed into and amplified in *E. coli* for DNA sequencing. Notably, 8 out of 40 samples resulted in sequencing failure and were not included for diversity analysis.

|  | gRNA1 | | gRNA2 | |
| --- | --- | --- | --- | --- |
| No. | AID | Target | AID | Target |
| 1 | A | *TIS11* | A | *MUM3* |
| 2 | A | *UFO1* | I | *3450* |
| 3 | I | *YMC1* | I | *GAL3* |
| 4 | D | *MAN2* | D | *OCA4* |
| 5 | I | *4827* | A | *NAR1* |
| 6 | I | *SWT21* | A | *ICS3* |
| 7 | I | *EHD3* | A | *SLG1* |
| 8 | I | *NUP157* | I | *PAU11* |
| 9 | D | *UBC8* | A | *PAU9* |
| 10 | I | *2058* | D | *SKI7* |
| 11 | D | *ECM22* | D | *TFB3* |
| 12 | I | *UBP6* | A | *PPZ2* |
| 13 | D | *PRM8* | A | *4333* |
| 14 | A | *MDJ2* | A | *PIF1* |
| 15 | I | *4304* | D | *ROT1* |
| 16 | I | *SEC61* | D | *AXL2* |
| 17 | D | *PUS5* | I | *SMX3* |
| 18 | I | *TDH3* | I | *ART10* |
| 19 | I | *WWM1* | D | *FAD1* |
| 20 | I | *DSE2* | D | *SME1* |
| 21 | A | *ITS1-2* | D | *MRPL6* |
| 22 | A | *MNN11* | A | *RUF23* |
| 23 | I | *TSC11* | A | *PRM3* |
| 24 | I | *MET14* | I | *MRX4* |
| 25 | I | *SCM4* | D | *4289* |
| 26 | A | *EST2* | I | *SEC14* |
| 27 | A | *NUT1* | I | *YAF9* |
| 28 | I | *AIR2* | A | *4786* |
| 29 | I | *ELM1* | A | *UFD2* |
| 30 | D | *YPS3* | A | *PMI40* |
| 31 | A | *5831* | I | *1395* |
| 32 | I | *ATG12* | D | *CDC43* |

**Supplementary Table 9.** Plasmids constructed in this study.

| Name | Description | Applications |
| --- | --- | --- |
| pAID6[^4^](#_ENREF_4) | pRS41K-INT-[dLbCpf1-VP]-Csy4-[dSpCas9-RD1152]-SaCas9 | Integrate AID |
| p426*-LbSgH[^4^](#_ENREF_4) | SNR52p-Scaffold-BsaI-BsaI-SUP4t cloned into *Bsa*I-free pRS426 | Helper plasmids for gRNA cloning |
| p426*-SpSgH[^4^](#_ENREF_4) | SNR52p-BsaI-BsaI-Scaffold-SUP4t cloned into *Bsa*I-free pRS426 |  |
| p426*-SaSgH[^4^](#_ENREF_4) | SNR52p-BsaI-BsaI-Scaffold-SUP4t cloned into *Bsa*I-free pRS426 |  |
| pSg482 | SPC97a guide sequences cloned into p426*-LbSgH | *SPC97a* |
| pSg483 | BUD22a guide sequences cloned into p426*-LbSgH | *BUD22a* |
| pSg486 | SIZ1i guide sequences cloned into p426*-SpSgH | *SIZ1i* |
| pSg487 | SLX5i guide sequences cloned into p426*-SpSgH | *SLX5i* |
| pSg488 | NUP133i guide sequences cloned into p426*-SpSgH | *NUP133i* |
| pSg489 | GPI17i guide sequences cloned into p426*-SpSgH | *GPI17i* |
| pSg490 | UME1i guide sequences cloned into p426*-SpSgH | *UME1i* |
| pSg553 | MRPL32a guide sequences cloned into p426*-LbSgH | *MRPL32a* |
| pSg554 | ASE1a guide sequences cloned into p426*-LbSgH | *ASE1a* |
| pSg558 | RCF1a guide sequences cloned into p426*-LbSgH | *RCF1a* |
| pSg591 | NAT1a guide sequences cloned into p426*-LbSgH | *NAT1a* |
| pSg592 | NRT1a guide sequences cloned into p426*-LbSgH | *NRT1a* |
| pSg593 | COQ4a guide sequences cloned into p426*-LbSgH | *COQ4a* |
| pSg549 | NEO1i guide sequences cloned into p426*-SpSgH | *NEO1i* |
| pSg587 | YNL146Wi guide sequences cloned into p426*-SpSgH | *YNL146Wi* |
| pSg588 | tH(GUG)Ki guide sequences cloned into p426*-SpSgH | *tH(GUG)Ki* |
| pSg589 | SNU66i guide sequences cloned into p426*-SpSgH | *SNU66i* |
| pSg590 | DDL1i guide sequences cloned into p426*-SpSgH | *DDL1i* |
| pSg615 | YNR064Ca guide sequences cloned into p426*-LbSgH | *YNR064Ca* |
| pSg616 | MGR1a guide sequences cloned into p426*-LbSgH | *MGR1a* |
| pSg617 | PEP7i guide sequences cloned into p426*-SpSgH | *PEP7i* |
| pSg618 | VPS8i guide sequences cloned into p426*-SpSgH | *VPS8i* |
| pSg619 | ZRT1i guide sequences cloned into p426*-SpSgH | *ZRT1i* |
| pSg621 | WHI2i guide sequences cloned into p426*-SpSgH | *WHI2i* |
| pSg622 | PDR1i guide sequences cloned into p426*-SpSgH | *PDR1i* |
| pSg624 | MUK1i guide sequences cloned into p426*-SpSgH | *MUK1i* |
| pFACS20 | 1^st^ round FACS isolated plasmid for HOC1 deletion | *HOC1d* |
| pFACS22 | 1^st^ round FACS isolated plasmid for UBP3 interference | *UBP3i* |
| pFACS23 | 1^st^ round FACS isolated plasmid for MNN9 interference | *MNN9i* |
| pFACS8 | 2^nd^ round FACS isolated plasmid for NUP157 interference | *NUP157i* |
| pFACS25 | 2^nd^ round FACS isolated plasmid for PDI1 activation | *PDI1a* |
| psMAGIC1 | Furfural tolerance screened plasmid for SFH1a-UBC9i |  |
| psMAGIC2 | Furfural tolerance screened plasmid for SIZ1d-SPC29i |  |
| psMAGIC3 | Furfural tolerance screened plasmid for SLX5i-SDS3i |  |
| pSg801 | SFH1a guide sequences cloned into p426*-LbSgH | *SFH1a* |
| pSg802 | UBC9i guide sequences cloned into p426*-SpSgH | *UBC9i* |
| pSg803 | NTH1d guide sequences cloned into p423*-SaSgH | *NTH1d* |
| pH5-TPS1 | *TPS1* overexpression cassette (*TEF1p-TPS1-TEF1t*) | *TPS1a* |
| pH5-ARI1 | *ARI1* overexpression cassette (*TEF1p-ARI1-TEF1t*) | *ARI1a* |
| pSg334 | X2-targeting guide sequences cloned into p426-SaSgH | SaCas9 mediated marker-less genome integration |
| pSg335 | X3-targeting guide sequences cloned into p426-SaSgH |  |
| pSg336 | X4-targeting guide sequences cloned into p426-SaSgH |  |
| pSg337 | XI1-targeting guide sequences cloned into p426-SaSgH |  |
| pSg338 | XI2-targeting guide sequences cloned into p426-SaSgH |  |
| pSg339 | XI3-targeting guide sequences cloned into p426-SaSgH |  |
| pSg340 | XI4-targeting guide sequences cloned into p426-SaSgH |  |
| pSg341 | XII2-targeting guide sequences cloned into p426-SaSgH |  |
| pSg342 | XII4-targeting guide sequences cloned into p426-SaSgH |  |
| pSg343 | XII5-targeting guide sequences cloned into p426-SaSgH |  |

**Supplementary Table 10.** Primers used in this study.

| Names | Sequences (5’-3’) | Applications |
| --- | --- | --- |
| X4-INT-T7F | ggtttccagccacagttgtagtcacgtgcgcgccatgctgtaatacgactcactataggg | Integrate EGII into X4 locus |
| X4-INT-T3R | cttggtagttggagcgcaattagcgtatcctgtaccatacaattaaccctcactaaaggg |  |
| LibA-F | tccttaagtggtccgtgttcggacctaatc | Amplify gRNA libraries from the oligo pools |
| LibA-R | ccagctgccacctctaagaatggacgacgt |  |
| LibI-F | cggagcagacattgtaaggctacgttcacc |  |
| LibI-R | gtaggcctctcgtgctatcttcgttggacg |  |
| LibD-F | gtatctcgcagccggtctccgatc |  |
| LibD-R | cggttctctctcgtggtctcgaaac |  |
| AID-NGS-F1 | tcgtcggcagcgtcagatgtgtataagagacagcttctccgcagtgaaagataaatgatc | Amplify gRNA libraries for NGS |
| AID-NGS-R1 | gtctcgtgggctcggagatgtgtataagagacagctttgagtgagctgataccgctcg |  |
| pSg482F | agatttgttccgcgactaccaggggaa | gRNA primers for SPC97a |
| pSg482R | aaaattcccctggtagtcgcggaacaa |  |
| pSg483F | agatatgagacgttttcttcattgatg | gRNA primers for BUD22a |
| pSg483R | aaaacatcaatgaagaaaacgtctcat |  |
| pSg486F | gatccagcagttccatcagagtga | gRNA primers for SIZ1i |
| pSg486R | aaactcactctgatggaactgctg |  |
| pSg487F | gatcagagcgtgtgttgcgttgat | gRNA primers for SLX5i |
| pSg487R | aaacatcaacgcaacacacgctct |  |
| pSg488F | gatcaaccaaaacatacaccattt | gRNA primers for NUP133i |
| pSg488R | aaacaaatggtgtatgttttggtt |  |
| pSg489F | gatcatacgtaacacagatttaac | gRNA primers for GPI17i |
| pSg489R | aaacgttaaatctgtgttacgtat |  |
| pSg490F | gatctcaacgcctgagccaaagat | gRNA primers for UME1i |
| pSg490R | aaacatctttggctcaggcgttga |  |
| pSg553F | agataggcaaagacaagaaaatacaag | gRNA primers for MRPL32a |
| pSg553R | aaaacttgtattttcttgtctttgcct |  |
| pSg554F | agatactaaataaccgcccagaaaatc | gRNA primers for ASE1a |
| pSg554R | aaaagattttctgggcggttatttagt |  |
| pSg558F | agatgatgcagacgtggccaagttggc | gRNA primers for RCF1a |
| pSg558R | aaaagccaacttggccacgtctgcatc |  |
| pSg591F | agatgacgcggagcagggtaaaaagtg | gRNA primers for NAT1a |
| pSg591R | aaaacactttttaccctgctccgcgtc |  |
| pSg592F | agatcccgaagaacaaatagcggtagc | gRNA primers for NRT1a |
| pSg592R | aaaagctaccgctatttgttcttcggg |  |
| pSg593F | agataggatgccgtaaaagaatgctcc | gRNA primers for COQ4a |
| pSg593R | aaaaggagcattcttttacggcatcct |  |
| pSg549F | gatcacagtgttatgcttactaag | gRNA primers for NEO1i |
| pSg549R | aaaccttagtaagcataacactgt |  |
| pSg587F | gatcaattaagattgtagagggag | gRNA primers for YNL146Wi |
| pSg587R | aaacctccctctacaatcttaatt |  |
| pSg588F | gatctacaacgtagaactgataaa | gRNA primers for tH(GUG)Ki |
| pSg588R | aaactttatcagttctacgttgta |  |
| pSg589F | gatctgaatacctataactgctaa | gRNA primers for SNU66i |
| pSg589R | aaacttagcagttataggtattca |  |
| pSg590F | gatctgtcgctttggaagaaaaag | gRNA primers for DDL1i |
| pSg590R | aaacctttttcttccaaagcgaca |  |
| pSg615F | agataatgactatgttaataacaaagg | gRNA primers for YNR064Ca |
| pSg615R | aaaacctttgttattaacatagtcatt |  |
| pSg616F | agattcattaaatagagatatataaga | gRNA primers for MGR1a |
| pSg616R | aaaatcttatatatctctatttaatga |  |
| pSg617F | gatccctttaaaaaccatgagatc | gRNA primers for PEP7i |
| pSg617R | aaacgatctcatggtttttaaagg |  |
| pSg618F | gatcggtgtaatgagtaatggtct | gRNA primers for VPS8i |
| pSg618R | aaacagaccattactcattacacc |  |
| pSg619F | gatcagatcatgacagccgatacc | gRNA primers for ZRT1i |
| pSg619R | aaacggtatcggctgtcatgatct |  |
| pSg621F | gatcctgttcttgtagaatcggag | gRNA primers for WHI2i |
| pSg621R | aaacctccgattctacaagaacag |  |
| pSg622F | gatcgcggccatatagacattacc | gRNA primers for PDR1i |
| pSg622R | aaacggtaatgtctatatggccgc |  |
| pSg624F | gatcgattgattagggtcaaacct | gRNA primers for MUK1i |
| pSg624R | aaacaggtttgaccctaatcaatc |  |
| pSg801F | agatgcatcttctgtaagtcagatgct | gRNA primers for SFH1a |
| pSg801R | aaaaagcatctgacttacagaagatgc |  |
| pSg802F | gatcagcataggataagcacacac | gRNA primers for UBC9i |
| pSg802R | aaacgtgtgtgcttatcctatgct |  |
| sMAGIC-F1 | nnnnnggtctccggactctttgaaaagataatgtatg | Amply gRNA cassettes to assemble the sMAGIC library |
| sMAGIC-R1 | nnnnnggtctccgctgcttgcatgcctgcagggagctc |  |
| sMAGIC-F1 | nnnnnggtctcccagctctttgaaaagataatgtatg |  |
| sMAGIC-R2 | nnnnnggtctcccaaccttgcatgcctgcagggagctc |  |
| qSIZ1-F | aacaattgccgaacattctggg | Primers for qPCR analysis |
| qSIZ1-R | tttcttggcgttggggatgata |  |
| qNAT1-F | atgatatcgagccatgcgtctt |  |
| qNAT1-R | cgcgtctttcaattgacccaat |  |
| qPDR1-F | ttcgatatcatctgcagggagc |  |
| qPDR1-R | aagggctgcggtaagtgattta |  |
| qNUP157-F2 | agtactagaaggggatgcaggt |  |
| qNUP157-R2 | taaaacgcctcttgactggtca |  |
| qACT1-F2 | ctgtcttcccatctatcgtcgg |  |
| qACT1-R2 | agcttcatcaccaacgtaggag |  |
| qSFH1-F | tggttgtggaatctttatccgg |  |
| qSFH1-R | tggtgttggtcttgcagatc |  |
| qUBC9-F | ccaaaggttaaatttccagccg |  |
| qUBC9-R | aagatcctgaaccccaagaac |  |
| Sg803-F | gatc caatctcgtatttgtaaagaa | gRNA primers for NTH1d |
| Sg803-R | aaac ttctttacaaatacgagattg |  |
| TPS1-F | nnnnnnggatcc atgactacggataacgctaagg | Amplify TPS1 coding sequences |
| TPS1-R | nnnnnnctcgag tcagtttttggtggcagaggag |  |
| ARI1-F | nnnnnnggatcc atgactactgataccactgttttc | Amplify ARI1 coding sequences |
| ARI1-R | nnnnnnctcgag ttaggcttcattttgaacttctaac |  |
| TPS1-INT-F | atgagtcaagttaatacaagccaaggaccggtagcccaag atagcttcaaaatgtttcta | Amplify TPS1 cassette |
| TPS1-INT-R | ctatagtccatagaggtttctttcttgaggccttaaactg atagcgccgatcaaagtatt |  |
| ARI1-INT-F | cagccacagttgtagtcacgtgcgcgccatgctgactaat atagcttcaaaatgtttcta | Amplify ARI1 cassette |
| ARI1-INT-R | tggtagttggagcgcaattagcgtatcctgtaccatacta atagcgccgatcaaagtatt |  |
| TPS1-I-ConF | caggagcgaggtacaagatttgttgg | Verification of TPS1 integration |
| TPS1-I-ConR | ggtattccaccccattaggcaatgtg |  |
| ARI1-I-ConF | cataagaggcaagaacaagcttc | Verification of ARI1 integration |
| ARI1-I-ConR | cttagataagttatgccaccagtg |  |
| pSg334F | gatcagtaagttgagtgtaaggtgg | gRNA for X2 integration |
| pSg334R | aaacccaccttacactcaacttact |  |
| pSg206F | gatcgtgattgttagttcagcgtaa | gRNA for X3 integration |
| pSg206R | aaacttacgctgaactaacaatcac |  |
|  |  |  |
| pSg207F | gatcggcagccgtcgttgggcagaa | gRNA for X4 integration |
| pSg207R | aaacttctgcccaacgacggctgcc |  |
| pSg337F | gatctgcatcgcgatgttagtttag | gRNA for XI1 integration |
| pSg337R | aaacctaaactaacatcgcgatgca |  |
| pSg338F | gatcccttctgttcatgcgtgacgg | gRNA for XI2 integration |
| pSg338R | aaacccgtcacgcatgaacagaagg |  |
| pSg339F | gatcggagaaaggaaagtagaaatg | gRNA for XI3 integration |
| pSg339R | aaaccatttctactttcctttctcc |  |
| pSg340F | gatcgtcgctaagatcattgtaact | gRNA for XII1 integration |
| pSg340R | aaacagttacaatgatcttagcgac |  |
| pSg341F | gatcaatagtctcacttactgggcg | gRNA for XII2 integration |
| pSg341R | aaaccgcccagtaagtgagactatt |  |
| pSg342F | gatctactgccacgtatttaatgag | gRNA for XII4 integration |
| pSg342R | aaacctcattaaatacgtggcagta |  |
| pSg343F | gatctctaccgtgagaaataaagca | gRNA for XII5 integration |
| pSg343R | aaactgctttatttctcacggtaga |  |
| X2-INT-F | gccacccataatcggcgcttagtttcggagttcaatcatactttgaaaagataatgtatg | Donor for X2 integration |
| X2-INT-R | atatggggtcagtggcgatattatactataggagttaaagaggaaacagctatgaccatg |  |
| X3-INT-F | atcaggcacgaaggcacactcgtatatgcatgttgttgaactttgaaaagataatgtatg | Donor for X3 integration |
| X3-INT-R | ttccatggggtcgcaacttttcccggtgacctctacatgtaggaaacagctatgaccatg |  |
| X4-INT-F | cagccacagttgtagtcacgtgcgcgccatgctgactaatctttgaaaagataatgtatg | Donor for X4 integration |
| X4-INT-R | tggtagttggagcgcaattagcgtatcctgtaccatactaaggaaacagctatgaccatg |  |
| XI1-INT-F | gcgccggttttcattttcttccacggaataccaagcccatctttgaaaagataatgtatg | Donor for XI1 integration |
| XI1-INT-R | ctgtacgcagcatttagcagagatttgccaatgccaagaaaggaaacagctatgaccatg |  |
| XI2-INT-F | ttcacgcaagttaagtccaggaaggtgagcaaatgctcatctttgaaaagataatgtatg | Donor for XI2 integration |
| XI2-INT-R | aggcacggaaacggctgcacgggtacgccagataaggataaggaaacagctatgaccatg |  |
| XI3-INT-F | ccaatcaaagaagcatcggttcagatcgagcaaactgtagctttgaaaagataatgtatg | Donor for XI3 integration |
| XI3-INT-R | tgacatccaaactacaaaaccgagattggacatatagcacaggaaacagctatgaccatg |  |
| XII1-INT-F | atacaatagcacatctcattacccagttatgattgacgtcctttgaaaagataatgtatg | Donor for XII1 integration |
| XII1-INT-R | cgaggaaaattagaattagtggagcaaataatgagcacagaggaaacagctatgaccatg |  |
| XII2-INT-F | tgcgtctaacgcttttgccacttggatttctattataggactttgaaaagataatgtatg | Donor for XII2 integration |
| XII2-INT-R | aagaaattcttcctgtgcttcatcaaaacgcgaaaattcgaggaaacagctatgaccatg |  |
| XII4-INT-F | agcgcttataaggttggggcaatactaaaactgtgatcttctttgaaaagataatgtatg | Donor for XII4 integration |
| XII4-INT-R | ttccgactctgttgtttcctattgtttctaatagggtacgaggaaacagctatgaccatg |  |
| XII5-INT-F | tttctaactcttctcacgctgcccctatctgttcttccgcctttgaaaagataatgtatg | Donor for XII5 integration |
| XII5-INT-R | ctagccttattgttttagttcagtgacagcgaactgccgtaggaaacagctatgaccatg |  |

**Supplementary Table 11.** Strains constructed in this study.

| Name | Genotypes |
| --- | --- |
| BY4742 | *MAT*α *his3*Δ1 *leu2*Δ0 *lys2*Δ0 *ura3*Δ0 |
| bAID | BY4742-Delta::KanMX-[*dLbCpf1-VP*]-[*Csy4*]-[*dSpCas9-RD1152*]-[*SaCas9*] |
| bAID-RV | bAID-X4::[*CYC1p-mCherry-TEF1t*]-[*TEF1p-mVenus-PGK1t*] |
| bAID-EG | bAID-X4::[*TEF1p-prepro-HIS-EGII-AGA1-PGK1t*] |
| R1 | bAID-X3::*SIZ1i* |
| R2 | bAID-X3::*SIZ1i*-X4::*NAT1a* |
| R3 | bAID-X3::*SIZ1i*-X4::*NAT1a*-XI1::*PDR1i* |
| T1 | Same as R1 |
| T2 | bAID-X4::*NAT1a* |
| T3 | bAID-XI1::*PDR1i* |
| T1+T2 | Same as R2 |
| T1+T3 | bAID-X3::*SIZ1i*-XI1::*PDR1i* |
| T2+T3 | bAID-X4::*NAT1a*-XI1::*PDR1i* |
| T1+T2+T3 | Same as R3 |
| EG11 | bAID-EG-*HOC1d* |
| EG12 | bAID-EG-*UBP3i* |
| EG13 | bAID-EG-*MNN9i* |
| EG21 | bAID-EG-*HOC1d*-*NUP157i* |
| EG22 | bAID-EG-*HOC1d*-*PDI1a* |
| ScTAΔN | bAID-NTH1::TPS1-X4::ARI1 |

**Supplementary Table 12.** NGS sequencing cassettes for CRISPRa, CRISPRi, and CRISPRd libraries. The 3’-end of SNR52 promoter sequences, SUP4 terminator sequences, and part of the vector sequences are shown in lower case, the gRNA structural sequences are capitalized, the guide sequences are represented as N, and the Illumina overhang adapter sequences were shown in green. The 43 bp region extracted from the NGS data for mapping into the reference sequences were highlighted in yellow.

|  | Sequences (5’ to 3’) |
| --- | --- |
| LibA | TCGTCGGCAGCGTCAGATGTGTATAAGAGACAGcttctccgcagtgaaagataaatgatcAATTTCTACTAAGTGTAGATNNNNNNNNNNNNNNNNNNNNNNNtttttttgttttttatgtctgagctccctgcaggcatgcaagcttggcgtaatcatggtcatagctgtttcctgtgtgaaattgttatccgctcacaattccacacaacatacgagccggaagcataaagtgtaaagcctggggtgcctaatgagtgagctaactcacattaattgcgttgcgctcactgcccgctttccagtcgggaaacctgtcgtgccagctgcattaatgaatcggccaacgcgcggggagaggcggtttgcgtattgggcgctcttccgcttcctcgctcactgactcgctgcgctcggtcgttcggctgcggcgagcggtatcagctcactcaaagCTGTCTCTTATACACATCTCCGAGCCCACGAGAC |
| LibI | TCGTCGGCAGCGTCAGATGTGTATAAGAGACAGcttctccgcagtgaaagataaatgatcNNNNNNNNNNNNNNNNNNNNGTTTTAGAGCTAGAAATAGCAAGTTAAAATAAGGCTAGTCCGTTATCAACTTGAAAAAGTGGCACCGAGTCGGTGCTTTTTTGATCCtttttttgttttttatgtctgagctccctgcaggcatgcaagcttggcgtaatcatggtcatagctgtttcctgtgtgaaattgttatccgctcacaattccacacaacatacgagccggaagcataaagtgtaaagcctggggtgcctaatgagtgagctaactcacattaattgcgttgcgctcactgcccgctttccagtcgggaaacctgtcgtgccagctgcattaatgaatcggccaacgcgcggggagaggcggtttgcgtattgggcgctcttccgcttcctcgctcactgactcgctgcgctcggtcgttcggctgcggcgagcggtatcagctcactcaaagCTGTCTCTTATACACATCTCCGAGCCCACGAGAC |
| LibD | TCGTCGGCAGCGTCAGATGTGTATAAGAGACAGcttctccgcagtgaaagataaatgatcNNNNNNNNNNNNNNNNNNNNNNNNNNNNNNNNNNNNNNNNNNNNNNNNNNNNNNNNNNNNNNNNNNNNNNNNNNNNNNNNNNNNNNNNNNNNNNNNNNNNNNNNNNNNNNNNNNNNNNNNNGTTTTAGTACTCTGTAATTTTAGGTATGAGGTAGACGAAAATTGTACTTATACCTAAAATTACAGAATCTACTAAAACAAGGCAAAATGCCGTGTTTATCTCGTCAACTTGTTGGCGAGATTTTTTTGATCCtttttttgttttttatgtctgagctccctgcaggcatgcaagcttggcgtaatcatggtcatagctgtttcctgtgtgaaattgttatccgctcacaattccacacaacatacgagccggaagcataaagtgtaaagcctggggtgcctaatgagtgagctaactcacattaattgcgttgcgctcactgcccgctttccagtcgggaaacctgtcgtgccagctgcattaatgaatcggccaacgcgcggggagaggcggtttgcgtattgggcgctcttccgcttcctcgctcactgactcgctgcgctcggtcgttcggctgcggcgagcggtatcagctcactcaaagCTGTCTCTTATACACATCTCCGAGCCCACGAGAC |

**SUPPLEMENTARY REFERENCES**

1 Bao, Z. *et al.* Genome-scale engineering of *Saccharomyces cerevisiae* with single-nucleotide precision. *Nat Biotechnol* **36**, 505-508 (2018).

2 Divate, N. R., Chen, G. H., Divate, R. D., Ou, B. R. & Chung, Y. C. Metabolic engineering of *Saccharomyces cerevisiae* for improvement in stresses tolerance. *Bioengineered* **8**, 524-535 (2017).

3 Labun, K., Montague, T. G., Gagnon, J. A., Thyme, S. B. & Valen, E. CHOPCHOP v2: a web tool for the next generation of CRISPR genome engineering. *Nucleic Acids Res* **44**, W272-276 (2016).

4 Lian, J., HamediRad, M., Hu, S. & Zhao, H. Combinatorial metabolic engineering using an orthogonal tri-functional CRISPR system. *Nat Commun* **8**, 1688 (2017).
