# Supplementary material for: Multi-functional genome-wide CRISPR system for high throughput genotype–phenotype mapping
Source: Nat Commun. 2019 Dec 19;10:5794. doi: 10.1038/s41467-019-13621-4 (PMC6923430; doi:10.1038/s41467-019-13621-4)
Supplement: Supplementary file 2 — Description of Additional Supplementary Files [file 41467_2019_13621_MOESM2_ESM.docx]

**Description of Additional Supplementary Files**

File Name: Supplementary Data 1
Description: A summary of the genome-scale CRISPRa guide sequences.

File Name: Supplementary Data 2
Description: A summary of the genome-scale CRISPRi guide sequences.

File Name: Supplementary Data 3
Description: A summary of the genome-scale CRISPRd guide sequences.

File Name: Supplementary Data 4
Description: Reference sequences.
